# Incidence of acute otitis media in children in the United States before and after the introduction of 7- and 13-valent pneumococcal conjugate vaccines during 1998–2018

**DOI:** 10.1186/s12879-022-07275-9

**Published:** 2022-03-26

**Authors:** Tianyan Hu, Nicolae Done, Tanaz Petigara, Salini Mohanty, Yan Song, Qing Liu, Esteban Lemus-Wirtz, James Signorovitch, Eric Sarpong, Thomas Weiss

**Affiliations:** 1 Merck & Co., Inc., 2000 Galloping Hill Road, Kenilworth, NJ 07033 USA; 2 Analysis Group, Inc., Boston, MA USA

**Keywords:** Acute otitis media, United States, Pneumococcal conjugate vaccine, Healthcare utilization, Incidence, Epidemiology

## Abstract

**Background:**

Acute otitis media (AOM) is a leading cause of office visits and antibiotic prescriptions in children. Pneumococcal conjugate vaccines were introduced in the USA in 2000 (7-valent, PCV7) and 2010 (13-valent, PCV13). Expanded valency PCVs are currently under development. To describe the impact of PCVs and quantify the residual burden of AOM, this study estimated annual incidence rates (IRs) of AOM and AOM-related complications and surgical procedures in children < 18 years in the USA before and after the introduction of PCV7 and PCV13.

**Methods:**

AOM episodes were identified in the IBM MarketScan^®^ Commercial and Medicaid databases using diagnosis codes (ICD-9-CM: 382.x; ICD-10-CM: H66.xx and H67.xx). Annual IRs were calculated as the number of episodes per 1000 person-years (PYs) for all children < 18 years and by age group (< 2, 2–4, and 5–17 years). National estimates of annual AOM IRs were extrapolated using Census Bureau data. Interrupted time series analyses were used to assess immediate and gradual changes in monthly AOM IRs, controlling for seasonality.

**Results:**

In the commercially insured population, AOM IRs declined between the pre-PCV7 period (1998–1999) and the late PCV13 period (2014–2018) from 1170.1 to 768.8 episodes per 1000 PY for children < 2 years, from 547.4 to 410.3 episodes per 1000 PY in children 2–4 years, and from 115.6 to 91.8 episodes per 1000 PY in children 5–17 years. The interrupted time series analyses indicated significant immediate or gradual decreases in the early PCV7 period (2001–2005), and gradual increases in the late PCV7 period (2006–2009) in children < 2 years; however, crude IRs trended downward in all time periods. In older children, IRs decreased in the early PCV7 and early PCV13 period (2011–2013), but gradually increased in the late PCV7 period. IRs of AOM-related surgical procedures decreased, and IRs of AOM-related complications increased during the study timeframe.

**Conclusions:**

AOM disease burden remains high in children of all ages despite overall reductions in AOM IRs during 1998–2018 following the introduction of PCV7 and PCV13. The impact of investigational PCVs on the disease burden of AOM will likely depend on AOM etiology and circulating pneumococcal serotypes.

**Supplementary Information:**

The online version contains supplementary material available at 10.1186/s12879-022-07275-9.

## Background

Acute otitis media (AOM) is characterized by middle ear effusion, inflammation of the tympanic membrane, and the rapid onset of signs and symptoms of an acute infection [1, 2]. Approximately 23% of children in the United States (USA) experience an AOM episode before the age of one and about 60% of children experience an AOM episode before the age of three [3]. AOM is a leading cause of physician office visits and antibiotic prescriptions in children and is associated with medical expenditures of about $4 billion annually in the USA [4].

*Streptococcus pneumoniae* is a leading bacterial cause of AOM [3, 5, 6]. In 2000, a 7-valent pneumococcal conjugate vaccine (PCV7) was licensed in the US, and recommended for routine use in children by the US Advisory Committee on Immunization Practices (ACIP) [7]. In 2010, a 13-valent PCV (PCV13), which included six additional serotypes, was recommended by the ACIP and replaced PCV7 [7, 8].

To date, several retrospective studies using surveillance and administrative claims data have shown reductions in the incidence and costs attributable to AOM following the introduction of PCV7 and PCV13 [8–13]. A study by Zhou et al. found that ambulatory otitis media (OM) visit rates, including AOM and OM with effusion (OME), decreased from 2173 during 1997–1999 to 1244 visits per 1000 person-years (PY) in 2004 among children < 2 years [9]. Another study by Kawai et al. found that ambulatory OM visit rates decreased significantly after the introduction of PCV7 across all age groups (< 2, 2–4, 5–18), with the highest declines among children < 2 years [11]. Multiple other studies have also documented decreases in AOM incidence in children after the introduction of PCV13 [8, 10, 11, 13]. One exception was a 2018 study by Tong et al., which found a 13.5% significant decline in AOM incidence among the youngest children (< 2 year of age), with neglible changes in incidence among older children between 2008 and 2014 [14].

Only one previous study, however, has examined PCV impact in the USA up to 2016, and none have assessed the impact of both PCV7 and PCV13 comprehensively in a single analysis [8]. In addition, no study has examined the impact of PCVs on simple versus recurrent AOM, which may have different implications on the clinical and economic burden of disease. New vaccines are currently under development for the prevention of pneumococcal disease, including AOM [15–17]. These investigational vaccines contain all serotypes in the currently licensed PCV13 as well as additional serotypes. To better understand the current burden of AOM and the potential value of new vaccines, it is important to quantify the incidence of AOM following the introduction of PCV7 and PCV13 and the residual burden that remains prior to the introduction of higher valent PCVs.

This study estimated incidence and trends in incidence for all AOM, simple and recurrent AOM, as well as AOM-related complications and surgical procedures among children aged < 18 years in the USA before and after the introduction of PCV7 and PCV13.

## Methods

This study used data from the IBM MarketScan® Commercial Claims and Encounters (CCAE; January 1, 1998 to December 31, 2018) and Multi-State Medicaid databases (January 1, 2001 to December 31, 2018). The CCAE database contains enrollment eligibility, demographic characteristics, and medical, surgical, and prescription drug utilization and expenditure data for approximately 90 million employees and dependents covered by employer-sponsored private health insurance. The Multi-State Medicaid Databases contain the same data for nearly 16 million Medicaid enrollees from 12 states.^1^ Both databases contain information on inpatient, outpatient, and long-term care services.

### Study design and patient population

This was a retrospective observational cohort study of administrative claims data from USA children < 18 years enrolled in commercial or Medicaid plans at any time during the study period. Children with an AOM episode, defined using International Classification of Diseases 9/10th Revision, Clinical Modification (ICD-9-CM and ICD-10-CM) codes, were identified in each calendar year. Five periods were evaluated: (1) pre-PCV7 [1998–1999]; (2) early PCV7 [2001–2005]; (3) late PCV7 [2006–2009]; (4) early PCV13 [2011–2013]; (5) late PCV13 [2014–2018]. The years 2000 and 2010 were considered transitional years, when the PCV7 and PCV13 vaccines were introduced, respectively.

### Study outcomes

#### AOM episodes

AOM episodes were identified through inpatient and outpatient claims by the presence of a diagnosis code of suppurative or unspecified OM (ICD-9-CM: 382.x [13]; ICD-10-CM: H66.xx and H67.xx [18]). These diagnosis codes were identified in the primary positions of inpatient claims and in the primary or secondary positions of outpatient claims. An AOM episode was defined as one or more outpatient or inpatient claims; a gap of at least 14 days with no claims with AOM-related diagnoses was required to define the start of a new episode [19]. The service date of the first claim during each episode was assigned as the index date, and the index date was used to assign episodes to a calendar year. Episodes that crossed calendar years were assigned to the year in which the episode began. An AOM episode was categorized as recurrent if a patient had (1) three or more episodes within a 6-month period or (2) four or more episodes within a 12-month period, with at least one episode in the preceding 6 months, based on clinical guidelines [20]. The period of time examined for the calculation of recurrent episodes was thus 12 months prior to the year of analysis, or from the presumed birth month for patients aged < 1 year, in order to adequately capture prior episodes.

#### AOM-related complications and surgical procedures

AOM-related complications such as tympanic membrane perforation, otorrhea, and acute mastoiditis were identified using ICD-9/ICD-10-CM diagnosis codes observed within 21 days of the index date of each AOM episode [10]. AOM-related surgical procedures such as myringotomy and ventilation tube insertion occurring any time during the calendar year were identified using ICD-9/10-CM procedure codes and Current Procedure Terminology (CPT) codes [10] (the full list of diagnosis and procedure codes used in the study is shown in Additional file 1: Table A1. Procedures with the same code occurring on the same day were only counted once.

### Statistical analysis

Crude annual incidence rates (IRs) for AOM episodes were calculated by dividing the total number of AOM episodes by the total number of PY of health plan enrollment for all children in the corresponding calendar year and were expressed as episodes per 1000 PY. As the exact date of birth was not available, age was imputed assuming July 1 as the birthdate in each study year. IRs were calculated separately for simple and recurrent AOM episodes. IRs for AOM-related complications and AOM-related surgical procedures were similarly calculated.

Average IRs were calculated for each of the five time periods of interest, both for the overall population and stratified by age group, sex, region (i.e., Northeast, North Central, South, West, East), and urbanicity (i.e., urban or rural designation based on Metropolitan Statistical Area of residence of the primary beneficiary) for the commercially insured population, and by age group and sex for the Medicaid population (see Additional file 1: Appendix for Medicaid results). National estimates of annual IRs were calculated via direct standardization of crude annual IRs in the MarketScan population by age, sex, and insurance type (commercial vs. Medicaid) using Census Bureau data for each study year (additional details can be found in the Additional file 1: Appendix).

### Interrupted time series analyses

Interrupted time series (ITS) analyses were conducted to assess changes in the temporal trends of IRs before and after the introduction of PCV7 and PCV13. The ITS analyses were performed using generalized linear models (GLMs) with a negative binomial distribution and log link. Specifically, the AOM episode counts in each month were modeled in a segmented regression framework, with the number of enrolled children in each month included as an offset term. For the commercially insured population model, monthly AOM IRs in the early PCV7, late PCV7, early PCV13, and late PCV13 periods were each compared with those in the previous period. For each period, adjusted incidence rate ratios (IRRs) and 95% confidence intervals (95% CIs) were estimated for changes in level (i.e., an immediate change in IRs compared to the previous period) and changes in trend (i.e., a gradual change in IRs over time compared to the trends in the previous period). Models adjusted for seasonal fluctuations in IRs by using monthly indicators.

Separately, ITS analyses were conducted for the Medicaid population between 2006 and 2018. Due to the lack of Medicaid data during the pre-PCV7 period in this database, the Medicaid analyses assessed changes in IRs in the early and late PCV13 periods compared to the late PCV7 years. Models also adjusted for seasonality (see Additional file 1: Appendix for additional details).

Linear trends associated with each PCV vaccine period were assessed based on the predicted IRs from the negative binomial models, and presented graphically. All ITS analyses were conducted for children < 18 years, and separately by age groups (< 2, 2–4, and 5–17 years). Statistical analyses were conducted using SAS version 9.4 (SAS Institute, Inc., Cary, NC, USA) and R statistical software (R Foundation for Statistical Computing, Vienna, Austria).

### Sensitivity analyses

Two sensitivity analyses were conducted with alternative definitions of disease episodes. In the first sensitivity analysis, to explore the sensitivity of results to the gap definition, a gap of at least 28 days with no AOM-related diagnoses was used to define the start of a new episode (see Additional file 1: Appendix) [14].

In the second sensitivity analysis, a broader definition of OM was used, which included both AOM and OME. OME was defined by the presence of diagnosis codes for nonsuppurative OM and Eustachian tube disorder (ICD-9-CM: 381.x ICD-10-CM: H65.xx, H68.xx, H69.xx), or acute myringitis without mention of OM (ICD-9-CM: 384.0x; ICD-10-CM: H73.0x). This analysis was conducted to reflect revised diagnostic criteria issued by the American Academy of Pediatrics in 2013 to better discriminate between AOM and OME and reduce unnecessary antibiotic prescriptions [20]. This definition is consistent with some previously published studies, which typically analyzed AOM and OME together as OM [10]. In this sensitivity analysis, OM episodes were defined using a gap of at least 28 days between consecutive claims with relevant diagnosis codes (see Additional file 1: Appendix).

## Results

### Demographic characteristics and risk factors

Over the timeframe of the study, on average, 12.49 million commercially insured children aged 0–17 years contributed 5.81 million PY at risk each year. Sample sizes for populations at risk by study year are shown in the Additional file 1: Table A2 for the commercially insured and Additional file 1: Table A3 for the Medicaid population). Demographic characteristics for the populations at risk by study period are shown in Additional file 1: Table A4.

On average, approximately 1.21 million AOM episodes occurred annually in commercially insured children. Demographic characteristics for this population, for each of the study periods, are shown in Table 1. The proportion of patients aged < 2 years ranged from 28.6% to 31.5% across the study periods. Slightly more than half of patients (52.1%) were male, 36.7% to 48.7% lived in the South, and most lived in urban areas (70.3–83.2%). Over the timeframe of the study, the proportion of patients in fee-for-service plans declined considerably, while the proportion of patients enrolled in preferred provider organization/point of service and consumer directed health plans/high-deductible health plans increased. Demographic characteristics for Medicaid patients are shown in Additional file 1: Table A5.

**Table 1:** Demographic characteristics of commercially insured children aged < 18 years with AOM episodes, by PCV period (1998–2018)

Period^a^	Pre-PCV7	Early PCV7	Late PCV7	Early PCV13	Late PCV13
	(1998–1999)	(2001–2005)	(2006–2009)	(2011–2013)	(2014–2018)
Total number of children with AOM, N^b^	237,100	2,388,239	4,226,586	4,320,277	4,157,822
Age, mean (SD)^c,d^	4.82 (4.56)	4.63 (4.44)	4.65 (4.43)	4.89 (4.49)	4.93 (4.58)
< 2 years, %	31.1%	31.5%	30.9%	28.6%	29.2%
2–4 years, %	26.7%	28.3%	28.7%	28.6%	28.2%
5–17 years, %	42.2%	40.2%	40.4%	42.8%	42.6%
Male, %	51.8%	52.0%	52.1%	52.1%	52.1%
Region					
Northeast, %	17.7%	10.5%	10.7%	17.4%	17.5%
North Central, %	26.3%	23.9%	27.2%	25.9%	22.1%
South, %	40.4%	43.1%	48.7%	36.7%	44.4%
West, %	6.0%	21.1%	12.8%	17.1%	14.8%
Missing/unknown, %	9.6%	1.5%	0.6%	2.9%	1.2%
Urbanicity					
Urban, %	70.3%	80.8%	83.2%	82.7%	83.1%
Rural, %	20.0%	17.8%	16.2%	14.3%	11.9%
Missing, %	9.6%	1.4%	0.6%	2.9%	4.9%
Health plan types					
HMO/EPO, %	10.6%	23.2%	16.2%	14.3%	10.2%
PPO/POS, %	57.1%	66.4%	76.0%	69.4%	64.5%
CDHP/HDHP, %	0.0%	1.2%	2.8%	9.0%	20.5%
FFS, %	31.9%	6.6%	1.6%	0.9%	1.3%
Missing, %	0.4%	2.6%	3.3%	6.3%	3.5%

*AOM* acute otitis media, *CDHP* consumer directed health plan, *EPO* exclusive provider organization, *FFS* fee-for-service, *HDHP* high-deductible health plan, *HMO* health maintenance organization, *PCV* pneumococcal conjugate vaccine, *POS* point of service, *PPO* preferred provider organization, *SD* standard deviation

^a^Patients' demographic characteristics and risk factors were firstly determined by each calendar year and then combined by PCV periods, assuming each year has a distinct patient population

^b^For each calendar year, patients' demographic characteristics were determined at the index episode, which was defined as the first AOM episode in the given calendar year

^c^Patients' month and day of birth was imputed as July 1st for all patients. Age at onset was calculated as the difference between condition start date and imputed birth date

^d^Standard deviations for age in each vaccine period were calculated using the pooled standard deviation of the samples in relevant years

IRs of risk factors for pneumococcal disease in the 6 months prior to AOM episodes are shown in Additional file 1: Table A6. The most common risk factor was chronic lung disease including asthma, present on average across the study periods in 1.9% to 2.7% of commercially insured children with AOM. The second most common risk factor was cancer and iatrogenic immunosuppression, including radiotherapy, present in 0.9% to 1.5% of children.

### Incidence of AOM

#### Crude AOM IRs

**Fig. 1:**
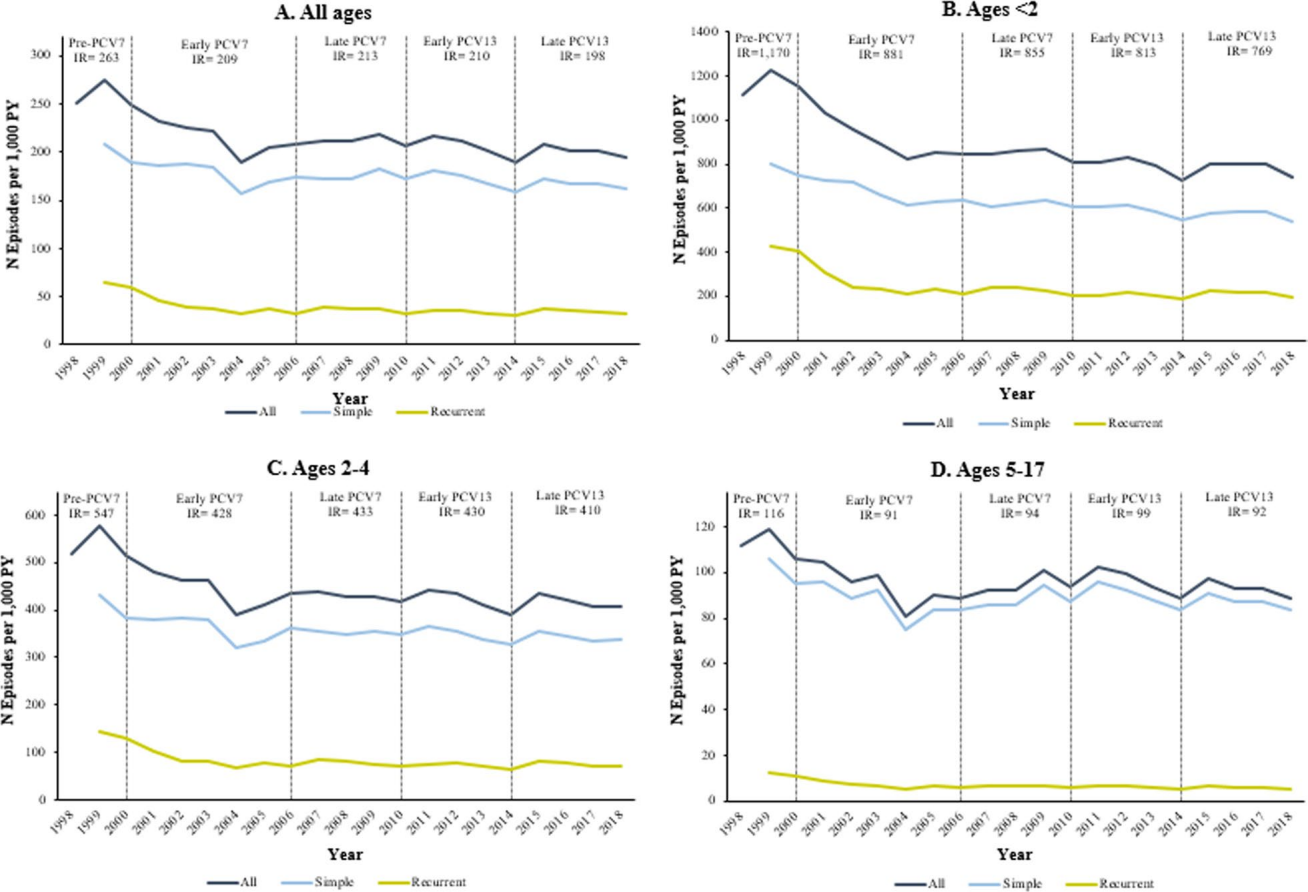
Trends in annual incidence rates of AOM by age group among commercially insured children aged < 18 years, in episodes per 1000 patient-years (1998–2018). *AOM* acute otitis media, *PCV* pneumococcal conjugate vaccine, *PY* person-years. Simple and recurrent episodes were categorized in patients with at least 12 months of continuous health plan enrollment prior to the index episode

Annual IRs of overall, simple, and recurrent AOM for commercially insured children are presented graphically in time series plots in Fig. 1 (Medicaid results are presented in Additional file 1: Fig. A1) for all children < 18 years and by age group.

Average annual IRs for overall, simple, and recurrent AOM and 95% CIs are presented for commercially insured children in Table 2 (results for Medicaid children are presented in Additional file 1: Table A7). AOM IRs declined over time among children < 18 years, from 263.2 to 198.1 episodes per 1000 PY from the pre-PCV7 to the late PCV13 period. During this timeframe, simple AOM IRs decreased from 209.3 to 165.1 episodes per 1000 PY, while recurrent AOM IRs decreased from 65.4 to 33.0 episodes per 1000 PY.

**Table 2:** Incidence rates of AOM and 95% confidence intervals by PCV period among commercially insured children aged < 18 years, in episodes per 1000 patient-years (1998–2018)

	All ages^a^			Ages < 2		
Period^b^	Overall	Simple	Recurrent^c^	Overall	Simple	Recurrent
Pre-PCV7	263.2 (262.4; 264.0)	209.3 (208.3; 210.3)	65.4 (64.8; 66.0)	1170.2 (1164.3; 1176.2)	801.3 (794.5; 808.3)	426.6 (421.6; 431.6)
Early PCV7	209.4 (209.2; 209.7)	172.7 (172.5; 172.9)	36.7 (36.6; 36.8)	880.6 (879.1; 882.0)	647.9 (646.7; 649.2)	232.6 (231.9; 233.4)
Late PCV7	212.8 (212.6; 212.9)	175.8 (175.7; 176.0)	36.9 (36.9; 37.0)	854.9 (853.8; 856.0)	626.9 (626.0; 627.8)	228.0 (227.4; 228.6)
Early PCV13	210.0 (209.9; 210.2)	175.5 (175.4; 175.7)	34.5 (34.4; 34.6)	813.1 (812.0; 814.1)	602.5 (601.6; 603.5)	210.5 (210.0; 211.1)
Late PCV13	198.1 (197.9; 198.2)	165.1 (164.9; 165.2)	33.0 (33.0; 33.1)	768.8 (767.7; 769.8)	562.6 (561.8; 563.5)	206.1 (205.6; 206.7)

	Ages 2–4			Ages 5–17		
Period	Overall	Simple	Recurrent	Overall	Simple	Recurrent
Pre-PCV7	547.4 (544.2; 550.6)	433.0 (429.0; 436.9)	144.3 (142.0; 146.6)	115.6 (114.9; 116.2)	106.4 (105.5; 107.2)	12.7 (12.4; 13.0)
Early PCV7	427.8 (427.0; 428.6)	350.3 (349.5; 351.0)	77.5 (77.2; 77.8)	91.2 (91.0; 91.4)	84.6 (84.5; 84.8)	6.6 (6.5; 6.6)
Late PCV7	432.9 (432.3; 433.5)	354.9 (354.4; 355.5)	77.9 (77.7; 78.2)	93.9 (93.8; 94.1)	87.6 (87.5; 87.7)	6.4 (6.3; 6.4)
Early PCV13	430.0 (429.4; 430.6)	355.1 (354.5; 355.6)	75.0 (74.7; 75.2)	99.0 (98.8; 99.1)	92.3 (92.2; 92.5)	6.6 (6.6; 6.7)
Late PCV13	410.3 (409.7; 410.9)	338.4 (337.9; 339.0)	71.9 (71.6; 72.1)	91.8 (91.7; 91.9)	86.2 (86.1; 86.3)	5.6 (5.5; 5.6)

*AOM* acute otitis media, *PY* person-years

^a^Confidence intervals were calculated using the Pearson method

^b^Time periods are defined as follows: Pre-PCV7: 1998–1999; Early PCV7: 2001–2005; Late PCV7: 2006–2009; Early PCV13: 2011–2013; Late PCV13: 2014–2018. Years 2000 and 2010 are considered transition years and were excluded

^c^Recurrent AOM was defined as having three or more episodes within a 6 months period or 4 or more episodes within a 12 month period, with at least one episode in the preceding 6 months

Overall, simple, and recurrent AOM IRs were all highest in children aged < 2 years; in this age group, AOM IRs declined steadily from 1170.2 to 768.8 episodes per 1000 PY between the pre-PCV7 and late PCV13 periods. Simple and recurrent AOM IRs decreased from 801.3 to 562.6 and from 426.6 to 206.1 episodes per 1000 PY, respectively, during this timeframe. In children aged 2–4 years, AOM IRs declined from 547.4 to 410.3 per 1000 PY between the pre-PCV7 and late PCV13 periods, while recurrent AOM IRs declined from 144.3 to 71.9 episodes per 1000 PY. In children aged 5–17 years, AOM IRs declined from 115.6 to 91.8 per 1000 PY between 1998 and 2018, while recurrent episode IRs decreased from 12.7 to 5.6 episodes per 1000 PY between 1998 and 2018.

Annual AOM IRs for Medicaid children are presented in Additional file 1: Fig. A1. AOM IRs stratified by sex, region, and urbanicity in commercially insured children are shown in Additional file 1: Figs. A2–A4, and AOM IRs stratified by sex among Medicaid-insured children are shown in Additional file 1: Fig. A5.

### Results of ITS analyses

The estimated IRRs from the ITS analyses are summarized in Table 3. In children < 2 years, monthly IRs increased gradually during the pre-PCV7 period (IRR 1.007, 95% CI [1.002–1.013], *P* < 0.01). Compared to this period, there was a 13.3% immediate decrease (IRR 0.867, 95% CI [0.794–0.946], *P* < 0.01) and a 1.2% per month gradual decrease (IRR 0.988, 95% CI [0.983–0.994], *P* < 0.001]) in monthly IRs in the early PCV7 period. In the late PCV7 period, monthly IRs increased gradually in this subgroup compared to the early PCV7 period (IRR 1.005, 95% CI [1.003–1.007], *P* < 0.001). No significant immediate or gradual changes in monthly IRs occurred in children < 2 years in the early PCV13 period compared to the late PCV7 period, or in the late PCV13 period compared to the early PCV13 period.

**Table 3:** Incidence rate ratio estimates from ITS analyses of monthly AOM episode incidence rates in commercially insured children aged < 18 years (1998–2018)

		All ages		Ages < 2		Ages 2–4		Ages 5–17	
Period^a^	Change	IRR^b^ (95% CI)^c^	*P*^a^	IRR (95% CI)	*P*	IRR (95% CI)	*P*	IRR (95% CI)	*P*
Pre-PCV7	Base Trend	1.005 (1.003–1.008)	0.001*	1.007 (1.002–1.013)	0.009*	1.006 (1.003–1.010)	0.001*	1.003 (0.998–1.007)	0.271
Early PCV7	Change in Level	0.888 (0.822–0.959)	0.003*	0.867 (0.794–0.946)	0.001*	0.887 (0.807–0.975)	0.013*	0.929 (0.812–1.063)	0.284
	Change in Trend	0.991 (0.988–0.994)	0.001*	0.988 (0.983–0.994)	0.001*	0.990 (0.986–0.993)	0.001*	0.993 (0.989–0.998)	0.004*
Late PCV7	Change in Level	1.069 (0.982–1.164)	0.125	1.065 (0.976–1.162)	0.159	1.119 (1.029–1.218)	0.009*	1.044 (0.925–1.178)	0.488
	Change in Trend	1.005 (1.003–1.007)	0.001*	1.005 (1.003–1.007)	0.001*	1.004 (1.002–1.006)	0.001*	1.007 (1.004–1.011)	0.001*
Early PCV13	Change in Level	1.046 (0.965–1.134)	0.270	0.978 (0.854–1.120)	0.747	1.076 (0.996–1.163)	0.064	1.090 (0.973–1.221)	0.138
	Change in Trend	0.996 (0.993–0.998)	0.001*	0.998 (0.994–1.002)	0.374	0.997 (0.995–1.000)	0.030*	0.993 (0.990–0.996)	0.001*
Late PCV13	Change in Level	1.007 (0.936–1.083)	0.853	0.982 (0.904–1.068)	0.677	1.012 (0.936–1.094)	0.762	1.015 (0.921–1.118)	0.766
	Change in Trend	1.003 (1.000–1.007)	0.069	1.002 (0.997–1.006)	0.401	1.003 (1.000–1.007)	0.092	1.003 (0.999–1.008)	0.117

*AOM* acute otitis media, *IRR* incidence rate ratio, *ITS* interrupted time series, *PCV* pneumococcal conjugate vaccine

^a^Time periods are defined as follows: Pre-PCV7: 1998–1999; Early PCV7: 2001–2005; Late PCV7: 2006–2009; Early PCV13: 2011–2013; Late PCV13: 2014–2018. Years 2000 and 2010 are considered transition years and were excluded from the model

^b^All estimates were obtained through a negative binomial model with a log link, controlling for seasonality using monthly indicators. IRR’s are the exponentiated regression coefficients and represent a multiplicative change. Model intercepts not shown

^c^Confidence intervals have been adjusted for heteroscedasticity

^d^^∗^Coefficients statistically significant at *P* < 0.05

In children 2–4 years, monthly IRs also increased gradually during the pre-PCV7 period (IRR 1.006, 95% CI [1.003–1.010], *P* < 0.001). Compared to this period, in the early PCV7 period there was a 11.3% immediate decrease (IRR 0.887, 95% CI [0.807–0.975], *P* < 0.05) and a 1.0% per month gradual decrease (IRR 0.990, 95% CI [0.986–0.993], *P* < 0.001]) in monthly IRs. In the late PCV7 period, there was a 11.9% immediate increase (IRR 1.119, 95% CI [1.029–1.218], *P* < 0.01) and a 0.4% gradual increase (IRR 1.004, 95% CI [1.002–1.006], *P* < 0.001) in monthly IRs compared to the early PCV7 period.

In children 5–17 years, monthly IRs were relatively stable during the pre-PCV7 period. Compared to this period, there was a gradual decrease of 0.7% each month in IRs during the early PCV7 period (IRR 0.993, 95% CI [0.989–0.998], *P* < 0.01). In the late PCV7 period, monthly IRs gradually increased by 0.7% each month compared to the early PCV7 period (IRR 1.007, 95% CI [1.004–1.011], *P* < 0.001). In the early PCV13 period, monthly IRs gradually decreased in this subgroup by 0.7% each month compared to the late PCV7 period (IRR 0.993, 95% CI [0.990–0.996], *P* < 0.001). No significant immediate or gradual changes occurred during the late PCV13 period compared to the early PCV13 period. The predicted monthly IRs and linear trends are shown in Additional file 1: Fig. A6.

In the Medicaid population, the findings were generally similar, showing gradual increases in monthly IRs in all age groups in the late PCV7 period and gradual decreases in the early PCV13 period (Additional file 1: Table A8, Fig. A7). One exception is the late PCV13 period, during which the Medicaid monthly IRs showed an immediate decline in all age groups compared to the early PCV13 period.

### National estimates of AOM IRs

**Fig. 2:**
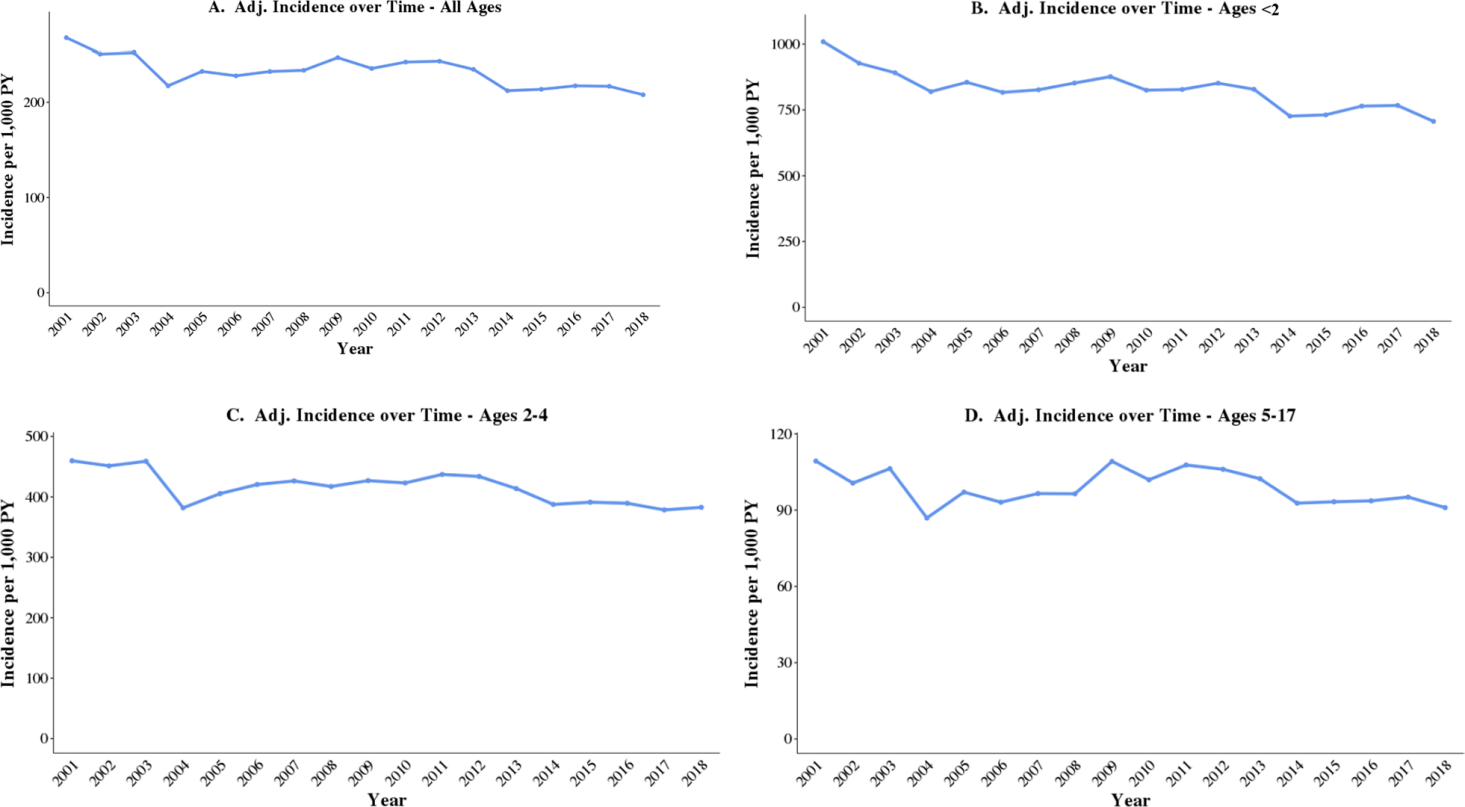
National estimates of annual AOM incidence rates episodes among USA children aged < 18 years (2001–2018). *Adj* adjusted, *AOM* acute otitis media, *PY* person-years. Incidence rates were adjusted using data from the U.S. Census Bureau, Current Population Survey and Annual Social and Economic Supplements. Census data for each study year were obtained from the USA Census Bureau database. Estimates of the July 1st USA population by sex, age, and insurance type were calculated for each study year by applying the average proportion of individuals with private and government health insurance for the 0–17 age group across all age-sex categories. AOM IRs in the general USA pediatric population were calculated by multiplying the IRs for each age-sex-insurance type group in the MarketScan data with the proportion of that group in the general USA pediatric population, and summing across all groups

Extrapolating results based on nationally available data, AOM IRs decreased from 268 episodes per 1000 PY in 2001 to 208 episodes per 1000 PY in 2018 (Fig. 2). Given these IRs and the overall pediatric population estimates in the US, there were approximately 3.80 million fewer AOM episodes projected in 2018 compared to 2001 among USA children (i.e. 19.47 million vs. 15.27 million episodes), a decrease of 21.6%.

### Incidence of AOM complications and surgical procedures

**Fig. 3:**
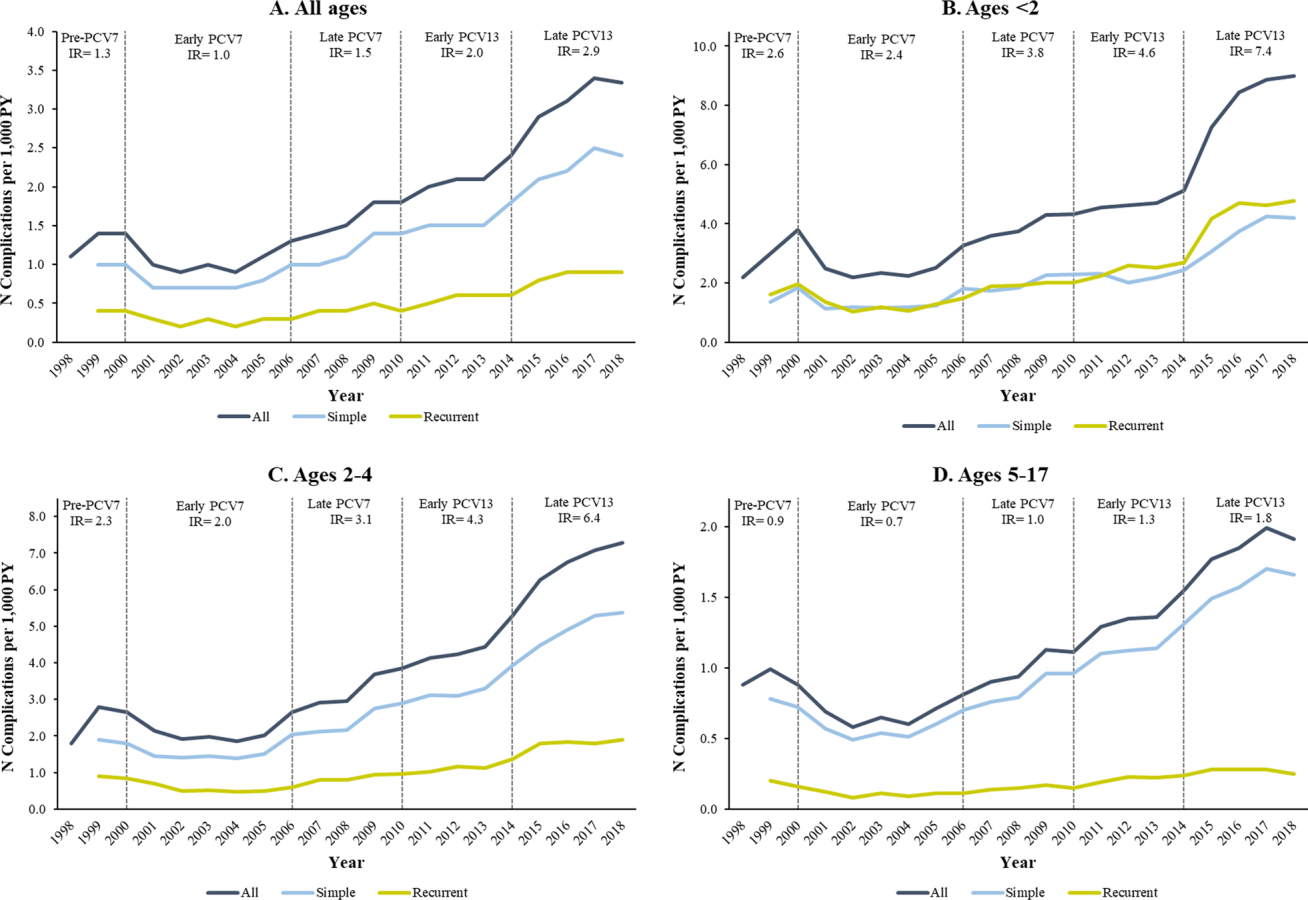
Annual incidence rates of AOM-related complications by age group among commercially insured children aged < 18 years, in complications per 1000 patient-years (1998–2018). *AOM* acute otitis media, *PY* person-years. Period-specific IRs are shown for total AOM-related complications

The incidence rate of AOM complications increased in children of all ages from 1.3 to 2.9 per 1000 PY between the pre-PCV7 and late PCV13 periods (Fig. 3A). Incidence increased from 2.6 to 7.4 per 1000 PY in children < 2 years (Fig. 3B), from 2.3 to 6.4 per 1000 PY in children 2–4 years (Fig. 3C) and from 0.9 to 1.8 per 1000 PY in children 5–17 years (Fig. 3D). Similar trends were observed in the Medicaid population (Additional file 1: Figs. A8, A9).

**Fig. 4:**
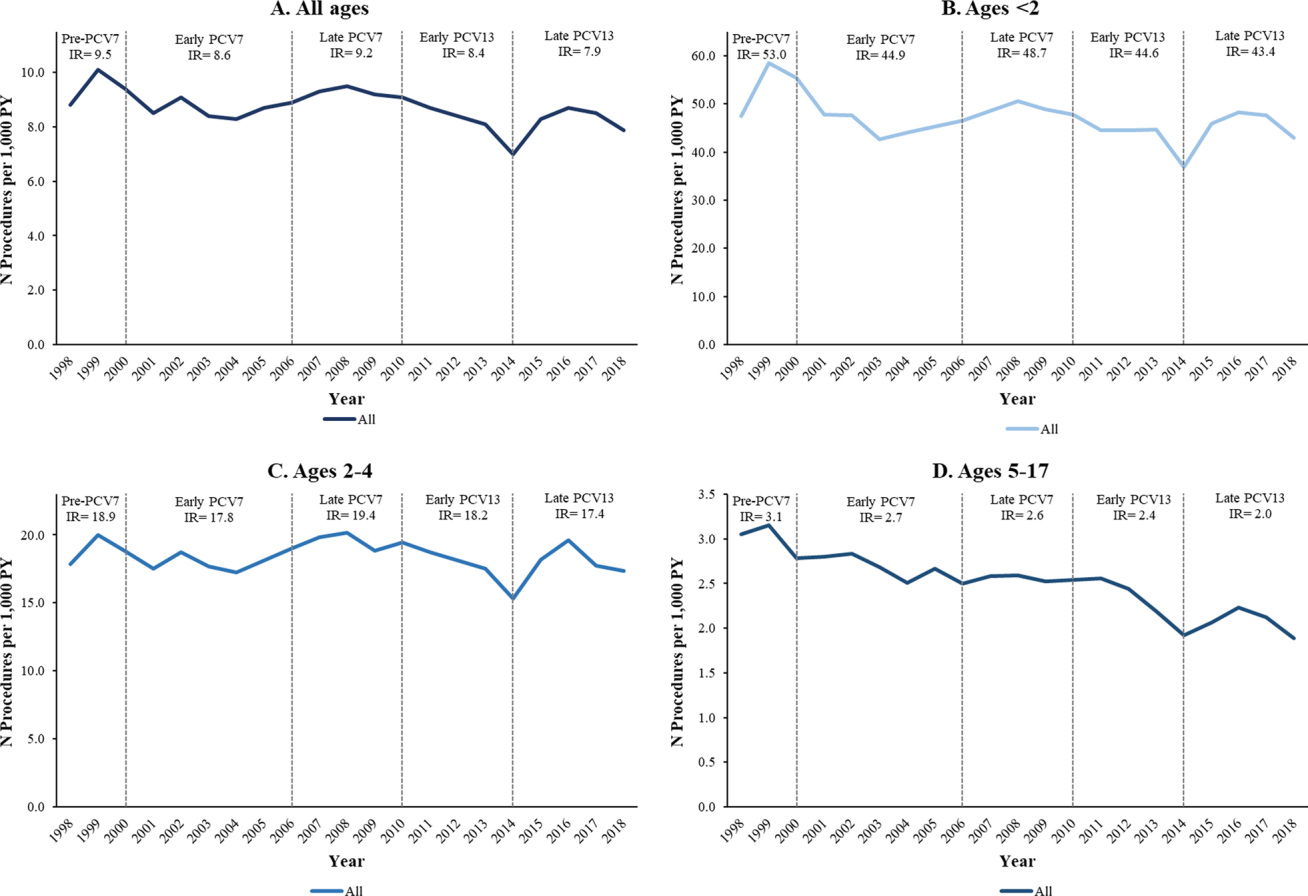
Annual incidence rates of AOM-related surgical procedures by age group among commercially insured children aged < 18 years, in procedures per 1000 patient-years (1998–2018). *AOM* acute otitis media, *PY* person-years

The rate of surgical procedures associated with AOM decreased slightly from 9.5 to 7.9 per 1000 PY between the pre-PCV7 and late PCV13 periods (Fig. 4A). Incidence decreased from 53.0 to 43.4 per 1000 PY in children < 2 years (Fig. 4B), from 18.9 to 17.4 per 1000 PY in children aged 2–4 years (Fig. 4C), and 3.1 to 2.0 per 1000 PY in children aged 5–17 years (Fig. 4D). Similar trends were observed in the Medicaid population (Additional file 1: Figs. A10, A11). Across study periods, a slight decline in surgical procedure IRs is apparent for the entire population, with smaller relative declines among patients aged < 2 and 2–4 years (Additional file 1: Fig. A11).

### Sensitivity analyses

The results of the sensitivity analyses are consistent with the main analysis. First, AOM IRs using a 28-day gap between consecutive claims to define disease episodes show similar trends over time, although the rates were lower overall (Additional file 1: Figs. A12, A13). Similarly, IRs of OM episodes show similar levels and trends over time, suggesting the results are robust to alternative outcome definitions (Additional file 1: Figs. A14, A15).

## Discussion

In this study of administrative claims data, we found that the incidence of AOM episodes decreased over time in commercially insured children overall, and in each of the three age groups examined. The interrupted time series analyses indicated significant immediate and gradual decreases during the early PCV7 period in children < 2 years; however, crude IRs showed a downward trend across all time periods. In older commercially insured children, IRs gradually decreased in the early PCV7 period, increased in the late PCV7 period, but gradually declined again in the early PCV13 period. The declines observed in the early PCV7 period were larger for younger children (i.e. aged < 2 and 2–4 years) compared to those in older children (aged 5–17 years), which is consistent with the PCV7 being targeted to younger children. Despite the overall reduction of AOM IRs in children < 18 years, our results suggest that rates stabilized in the late PCV13 period and that the residual burden of AOM in the US remains substantial, with an IR of 768.8, 410.3 and 91.8 per 1000 PY for children < 2 years, 2–4 years, and 5–17 years, respectively. The increase in the late PCV7 period could be caused, at least in part, by other AOM pathogens [21] or non-vaccine pneumococcal serotypes [22, 23]. Shifts in AOM etiology or pneumococcal serotype distribution as a result of PCV introduction can impact overall AOM incidence. Consistent with this explanation, prior studies have found that non-typable *Haemophilus influenzae* (NTHi) and *Moraxella catarrhalis* were the main pathogens replacing *S. pneumoniae* after the introduction of PCV7, with NTHi becoming the most common cause of AOM and recurrent AOM [3, 24–26]. Moreover, the non-vaccine serotype 19A that emerged as the most prevalent serotype in the PCV7 period was associated with increased virulence, multidrug resistance, and a propensity to cause complications such as mastoiditis, according to several studies in the USA [27–30]. Similar patterns are evident in Medicaid children, with the distinction that IRs remained relatively higher in this population during the late PCV7 and early PCV13 periods, before exhibiting a significant immediate reduction in the late PCV13 period in all age groups.

Despite differences in study methodology and data source which render cross-study comparisons challenging, our results are generally consistent with a moderate decline in AOM episodes, as reported by a number of previously published studies. For example, a 2018 claims-based study by Tong et al. [14], which also used the IBM MarketScan^®^ database, found that AOM IRs declined between 2008 and 2014 in children < 18 years but remained high at 431.8 per 1000 PY in year 2014. Tong et al. included nonsuppurative OM and Eustachian tube disorders (ICD-9-CM codes 381.x) in the AOM definition in addition to the code 382.x in this study, used a 28-day gap between claims to define episodes, and only analyzed the first episode during each year. Tong et al. suggested that the high AOM IR observed in their study may reflect minimal direct or indirect benefits from PCV13 that became available in 2010. Among children aged < 2 years, Tong et al. found a 13.5% decrease in AOM episodes similar to the 15.5% decrease in the current study during 2008–2014. This consistency in trends is confirmed by the fact that the proportion of code 382.x was 21%-24% of all AOM episodes during 1998–2015 (Additional file 1: Table A9), suggesting that the addition of code 381.x by Tong et al. did not affect the trends observed.

The decline in AOM episodes has also been reported in other studies [8, 10, 11]. For example, a 2018 study by Suaya et al. that used national projections from an audit of USA physicians found that among children 0–9 years, the IR of AOM declined by about 25% between 2011 and 2016, with similar declines in both the 0–2 and 3–9 year age group [8]. Our results are not directly comparable, given that Suaya et al. also used a narrower AOM definition based only on ICD-9-CM code 382.9 (unspecified OM) compared to 382.x (suppurative and unspecified OM) used in the current study. Given these differences, our observed declines in AOM IRs of 10% in children of all ages nationally, ranging from 8% in the < 2 year-old group, to 11% in the 2–4 year-old group, and 13% in the 5–17 year-old group, point to similar trends over time. While the decline in the youngest group is anticipated, the reasons for the decline in AOM rates among older children are less clear, particularly given the shortage in PCV7 nationally in the early 2000s [31].

The overall decline in AOM IRs is also consistent with findings from Marom et al. 2014, who detected a substantial decline in OM visit rates between 2004 and 2011 in a managed care population, including some Medicare and Medicaid patients [10]. Specifically, they found a decline of roughly 30% in IRs among children aged < 2, while the current study found a decline of 19.3% over the same period. However, in children aged 2–6 years they found a decline of 17.3%, while our results suggest no change in children aged 2–17 years. This difference in trends in older children can be explained by the fact that the population and the age groups have imperfect overlap. Similarly, using data from national surveys of medical care utilization, a study by Kawai et al. found declines in rates of OM-related physician office visits and hospital utilization among children 0–18 between 1997 and 2014, with the largest declines observed in younger children [11]. Although the decline in AOM IRs seen in our study and in these three prior studies can be partially attributed to PCV13, it may have also been confounded by the updated guidelines from the American Academy of Pediatrics [32], particularly changes in diagnostic criteria, which may have led to decreased diagnosis rates. However, discrepancies in the magnitudes of the declines across studies could be explained by differences in outcome definitions. For example, Marom et al. used the broader definition of OM, including the OME manifestation. Nevertheless, we found smaller declines in the PCV13 periods compared to those observed the PCV7 periods, consistent with studies that found other otopathogens such as *Moraxella catarrhalis*, as well as non-PCV13 serotypes of *S. pneumoniae*, predominantly 35B, 23B, and 15B/C, continued to grow during the PCV13 era, even as the highly virulent 19A serotype was nearly eliminated by the introduction of PCV13 [3, 33].

The results of the current analyses suggest that the majority of AOM episodes are simple AOM episodes; however, younger children are more likely to experience recurrent events than older children. This is consistent with a 2018 study by Kaur et al., which found that younger age was a strong predisposing characteristic for recurrent AOM episodes [3]. In the current study simple and recurrent AOM IRs have decreased in all age groups over time following the introduction of PCV7 and PCV13.

In the current study, IRs of AOM-related complications increased over time in all age groups. The finding is consistent with the study published in 2014 by Marom et al., which found an increase in certain OM-related complications such as tympanic membrane perforation/otorrhea (throughout 2001 to 2011) and mastoiditis (from 2001 to 2009) [10]. However, compared to Marom et al.’s estimated IRs which showed an increase from about 35 to 45 complications per 1000 PY between 2001 and 2011, our estimates are much lower, with IRs increasing from about 2.5 to 4.5 complications per 1000 PY during the same timeframe. A possible explanation is the narrower definition of AOM used in the current study (ICD-9 codes 382.x) compared to Marom et al. (381.x, 382.x, and 384.x) as well as the latter’s inclusion of code 384.x in the definition of OM, which likely leads to the identification of more visits with tympanic membrane perforation (384.2) in the 21-day period after the OM episode index date. Moreover, the current study reports the rate of AOM episodes that had any claims with complications which provides a more conservative measure of complications compared to counting all visits with such codes. However, neither the present study, nor the study by Marom et al., were able to assess the reasons for the increase in AOM-related complications beginning in 2004, which may include increasing rates of antibiotic resistance [10]. Evidence for decreasing rates of antibiotic susceptibility has also been documented for the PCV13 periods, likely caused by pneumococcal serotypes not included in PCV13 [34]. Another possible explanation, particularly for the more substantial increase in complication rates after 2013, may be an increasing shift to an observation-based management approach for AOM instead of antibiotic prescriptions, as recommended by the 2013 guidelines [5].

Results of the current study also indicated that IRs of AOM-related surgical procedures decreased overall (despite some fluctuations around 2000 and 2014). The decline in surgical procedures is consistent with the results in Marom et al., as well as previous and PCV7 efficacy trials, where PCV7 was demonstrated to be efficacious in reducing tympanostomy tube procedures within the first year after its introduction [35, 36]. The reduction in the frequency of OM observed following PCV administration was suggested as a contributing factor in the decline in OM-related procedures [35]. Although we observed reduction after the early PCV13 period, the pattern was mixed in the late PCV13 period, and the burden remains constant.

This study comprehensively investigated AOM IRs before and after the introduction of PCV7 and PCV13 from 1998 to 2018. However, the study has several limitations. First, as with most observational studies using claims databases, miscoding of diagnoses or procedures may occur, potentially leading to misclassification and measurement error. While it is possible for some of the sources of miscoding to remain relatively constant over time, other secular changes in clinical practice patterns or classification may also bias the results from the time-trends analyses. For example, changes from ICD-9-CM to ICD-10-CM diagnosis coding systems in 2015 may have affected the classification of diseases over time, potentially affecting the comparison of periods before vs. periods after this transition. Moreover, the current study examined AOM episodes due to all pathogens and all serotypes, being unable to identify the proportion of *S. pneumoniae*, and specific disease-causing serotypes from the claims database. Changes in clinical practice guidelines, parental behaviors, or prevalence of risk factors for AOM such as daycare attendance, breastfeeding, passive smoking, and influenza vaccination over time were not included in the study and may have also impacted trend comparisons. Moreover, an increase in enrollment for consumer-directed and high deductible health plans is evident over the timeframe of the study, from 0.0% in the pre-PCV7 period to 20.5% in the late PCV13 period; these plans incentivize less intensive care-seeking behavior, particularly for outpatient visits, which could have also contributed to declining trends in AOM episodes.

Second, an important limitation pertains to the representativeness of the IBM MarketScan data. Although the CCAE database is considered representative of commercial health plans in the US, it is based on non-random sampling of employer-sponsored health plans, with large employers over-represented. Thus, our results may not be generalizable to children with other types of commercial insurance. Similarly, the Medicaid databases only include data from a convenience sample of states. In addition, the data do not contain information on children without health insurance coverage. For these reasons, the national incidence rate estimates may not be fully representative of the entire USA population, and the potential for selection bias may be higher in earlier years due to the smaller cohort size available.

## Conclusions

The AOM disease burden remains substantial, despite overall reductions in AOM IRs following the introduction of PCV7 and PCV13 during the study period for children < 18 years, especially in children < 2 years. In older children, AOM IRs gradually increased in the late PCV7 period. Moreover, IRs of AOM-related complications increased, while IRs of AOM-related surgical procedures decreased over the timeframe of the study. Thus, further studies are warranted to confirm the current study findings using other data sources or study designs. The impact of future PCVs on AOM IRs will depend on the proportion of AOM caused by *S. pneumoniae* and vaccine-type serotypes.

## Supplementary Information

**Additional file 1.** Supplementary methods and results.

## Data Availability

The data that support the findings of this study are available from the IBM^®^ MarketScan^®^ Research Databases but restrictions apply to the availability of these data, which were used under license for the current study, and so are not publicly available. Data are however available from the authors upon reasonable request from the corresponding author with permission from IBM^®^ Watson Health™.

## Abbreviations

ACIP: Advisory Committee on Immunization Practices
AOM: Acute otitis media
CCAE: Commercial Claims and Encounters
CI: Confidence interval
CPT: Current procedure terminology
GLMs: Generalized linear models
ICD-9/10-CM: International Classification of Diseases 9/10th Revision, Clinical Modification
IRRs: Incidence rate ratios
IRs: Incidence rates
ITS: Interrupted time series
OM: Otitis media
OME: Otitis media with effusion
PCV13: 13-Valent pneumococcal conjugate vaccine
PCV7: 7-Valent pneumococcal conjugate vaccine
PYs: Person-years
US: United States

## Footnotes

1 Medicaid is a joint federal and state program that, along with the Children’s Health Insurance Program (CHIP), provides health and long-term coverage to low-income people in the United States. While eligibility rules vary by state, Medicaid typically covers children, pregnant women, elderly adults and people with disabilities, and eligible low-income adults. Medicaid is managed by each state and funded jointly by the federal and state governments. Many Medicaid enrollees receive care through private managed care plans contracted by the Medicaid program in their state. Because Medicaid programs in general pay lower rates than private health insurance plans, some providers (including hospitals) choose not to accept Medicaid patients or to accept only a limited number of such patients. However, in general the same providers can be accessible to both Medicaid and privately insured patients. Moreover, as a person’s income may change, they may lose (and subsequently regain) Medicaid eligibility, known as “churning.” Medicaid also tends to have a disproportionately larger share of black and Hispanic people compared to private plans.
