# Supplementary material for: Incidence of acute otitis media in children in the United States before and after the introduction of 7- and 13-valent pneumococcal conjugate vaccines during 1998–2018
Source: BMC Infect Dis. 2022 Mar 26;22:294. doi: 10.1186/s12879-022-07275-9 (PMC8962537; doi:10.1186/s12879-022-07275-9)
Supplement: Supplementary file 1 — Additional file 1. Supplementary methods and results. [file 12879_2022_7275_MOESM1_ESM.pdf]

Supplemental Appendix

Supplemental Table A1. Diagnosis and procedure codes used in the study

Condition	Categories	ICD-9-CM	ICD-10-CM	Descriptions
**OM**	AOM	382.x	H66.xxx - Suppurative and unspecified otitis media<br>H67.x - Otitis media in diseases classified elsewhere	Acute suppurative otitis media, suppurative and unspecified OM
	OME	381.x	H65.xxx - Nonsuppurative otitis media<br>H68.xxx - Eustachian salpingitis and obstruction<br>H69.xx - Other and unspecified disorders of Eustachian tube	Nonsuppurative otitis media and Eustachian tube disorders
		384.0x	H73.0x	Acute myringitis, unspecified
**AOM complications**		384.2	H72	Perforation of tympanic membrane, unspecified ear
		388.6	H92.1x/H92.2x	Otorrhea/otorrhagia
		383.0x	H70.0x	Acute mastoiditis
**AOM procedures**	ICD procedure codes	**ICD-9-CM Procedure Code:** 20.0x (myringotomy)	**ICD-10 Procedure Codes:** 099500Z, 099570Z, 099580Z, 099600Z, 099670Z, 099680Z, 099700Z, 099730Z, 099740Z, 099770Z, 099780Z, 099800Z, 099830Z, 099840Z, 099870Z, 099880Z	Drainage of Right/Left Middle Ear with Drainage Device, Open Approach<br>Drainage of Right/Left Middle Ear with Drainage Device, Via Natural or Artificial Opening<br>Drainage of Right/Left Middle Ear with Drainage Device, Via Natural or Artificial Opening Endoscopic<br>Drainage of Right/Left Tympanic Membrane with Drainage Device, Open Approach<br>Drainage of Right/Left Tympanic Membrane with Drainage Device, Percutaneous Approach<br>Drainage of Right/Left Tympanic Membrane with Drainage Device, Percutaneous Endoscopic Approach<br>Drainage of Right/Left Tympanic Membrane with Drainage Device, Via Natural or Artificial Opening<br>Drainage of Right/Left Tympanic Membrane with Drainage Device, Via Natural or Artificial Opening Endoscopic
	CPT codes		69420	Myringotomy including aspiration and/or Eustachian tube inflation
			69421	Myringotomy including aspiration and/or Eustachian tube inflation requiring general anesthesia
			69433	Tympanostomy requiring insertion of ventilating tube, with local or topical anesthesia
			69436	Tympanostomy requiring insertion of ventilating tube, with general anesthesia

Additional information on AOM episode definition

Only claims occurring during a patient's enrollment period were included. Claims were excluded if in addition to AOM diagnosis codes, they also had diagnosis codes for bacteremia, meningitis, or pneumonia, as these conditions were analyzed within the larger research project of which this study was part. This exclusion was performed in order to avoid attributing the health care encounters related to these more severe diseases commonly caused by *S. pneumoniae* to AOM. Overall, these exclusions are not likely to substantially affect the rates of AOM, given the much higher incidence of AOM relative to these diseases.

Derivation of national incidence rates

Data for each study year were obtained from the Census Bureau. Estimates of the July 1st US population by sex, age, and insurance type were calculated for each study year by applying the average proportion of individuals with private and government health insurance for the 0-17 age group across all age-sex categories. The AOM IRs in the general US pediatric population were calculated by multiplying the IRs for each age-sex-insurance type group in the MarketScan data with the proportion of that group in the general US pediatric population, and summing across all groups. This approach is equivalent to reweighting the IRs observed in each age-sex-insurance group based on population estimates of these groups in the US population. The main assumptions underlying this estimation are that the population in the MarketScan CCAE database is representative of the population with private insurance and that the MarketScan Medicaid databases are representative of the population covered by government insurance, after adjusting for age and sex, and that the proportion of US children without insurance coverage nationally is negligible. While not a random sample of the US population, the population covered by the MarketScan databases are large convenience samples of the US population that are broadly representative of healthcare seeking patterns nationally. Moreover, private insurance plans (mainly employer-sponsored) and Medicaid/CHIP represent the primary insurance coverage for over 98% of children in the US, which renders sensible the assumptions made in this analysis.

Interrupted time series estimation

Generalized linear models (GLM) with negative binomial family and log link function were used to estimate the parameters of the ITS models. The reasons for using GLM models are that they (1) offer flexibility when the outcome variable is constrained, in our case rates, or when errors are non-normally distributed, (2) are a recommended alternative to a log transformation of the outcome variable and (3) retain a similar analysis framework as linear models. The modified Park test was used in choosing the appropriate combination of distributional family and link function. A likelihood ratio test was used to test for overdispersion, and plots of standardized deviance residuals vs. the predicted model counts were examined, suggesting that the negative binomial model provides better fit.

The estimating equations used for each of the data sources are shown in detail below.

Equation used for the models estimated in the commercially insured population (1998-2018):

$$\ln(\mathrm{E}[N_i^{AOM}]) = \ln\left(N_i^{Risk}\right) + \beta_0 + \beta_1 T_i + \beta_2 \times EarlyPCV7_{\mathrm{i}} + \beta_3 \times TEarlyPCV7_{\mathrm{i}} + \beta_4 \times LatePCV7_{\mathrm{i}} + \beta_5 \times TLatePCV7_{\mathrm{i}} + \beta_6 \times EarlyPCV13_{\mathrm{i}} + \beta_7 \times TEarlyPCV13_{\mathrm{i}} + \beta_8 \times LatePCV13_{\mathrm{i}} + \beta_9 \times TLatePCV13_{\mathrm{i}} + \sum_{m=1}^{12} \gamma_m \times I_m$$

where:

N_i^{AOM} is the episode count in each population stratum for month i = 1 to n;

N_i^{Risk} is the population at risk in each population stratum for each month;

T_i is a linear time trend indicator, which equals 1 in January 1998 and increases by 1 with each subsequent month;

$EarlyPCV7$ is an indicator which equals 0 before January 2001 and 1 after the start of the early PCV7 period (i.e. from January 2001 onward);

$TEarlyPCV7$ is a linear time trend indicator for the early PCV7 period, which equals 0 before January 2001, then increases linearly each month as 1, 2, 3, … starting in January 2001;

$LatePCV7$ is an indicator which equals 0 before January 2006 and 1 after the start of the late PCV7 period (i.e. from January 2006 onward);

$TLatePCV7$ is a linear time trend indicator for the late PCV7 period, which equals 0 before January 2006 and then increases linearly each month as 1, 2, 3, … starting in January 2006;

$EarlyPCV13$ is an indicator which equals 0 before January 2011 and 1 after the start of the early PCV13 period (i.e. from January 2011 onward);

$TEarlyPCV13$ is a linear time trend indicator for the early PCV13 period, which equals 0 before January 2011 and then increases linearly each month as 1, 2, 3, … starting in January 2011;

$LatePCV13$ is an indicator which equals 0 before January 2014 and 1 after the start of the late PCV13 period (i.e. from January 2014 onward);

$TLatePCV13$ is a linear time trend indicator for the late PCV13 period, which equals 0 before January 2014 and then increases linearly each month as 1, 2, 3, … starting in January 2014;

I_m is an indicator for each month m = 2 to 12, i.e. February, … December; the indicator for January is excluded and serves as the reference.

Equation used for the Medicaid model (2006-2018):

$$\ln(\mathrm{E}[N_i^{AOM}]) = \ln\left(N_i^{Risk}\right) + \beta_0 + \beta_1 T_i + \beta_2 \times EarlyPCV13_{\mathrm{i}} + \beta_3 \times TEarlyPCV13_{\mathrm{i}} + \beta_4 \times LatePCV13_{\mathrm{i}} + \beta_5 \times TLatePCV13_{\mathrm{i}} + \sum_{m=1}^{12} \gamma_m \times I_m$$

where:

N_i^{AOM} is the episode count in each population stratum for month i = 1 to n;

N_i^{Risk} is the population at risk in each population stratum for each month;

T_i is a linear time trend indicator, which equals 1 in January 2006 and increases by 1 with each subsequent month;

$EarlyPCV13$ is an indicator which equals 0 before January 2011 and 1 after the start of the early PCV13 period (i.e. from January 2011 onward);

$TEarlyPCV13$ is a linear time trend indicator for the early PCV13 period, which equals 0 before January 2011 and then increases each month as 1, 2, 3, … starting in January 2011;

$LatePCV13$ is an indicator which equals 0 before January 2014 and 1 after the start of the late PCV13 period (i.e. from January 2014 onward);

$TLatePCV13$ is a linear time trend indicator for the late PCV13 period, which equals 0 before January 2014 and then increases each month as 1, 2, 3, … starting in January 2014;

I_m is an indicator for each month m = 2 to 12, i.e. February, … December; the indicator for January is excluded and serves as the reference;

Supplemental Table A2. Size of MarketScan commercially insured population at risk in person-years (1998-2018)

Year	Total Population at risk in person years			
	**All ages**	**Age 0-1**	**Age 2-4**	**Age 5-17**
1998	751,826	63,272	102,853	585,701
1999	773,498	64,818	105,906	602,773
2000	822,472	68,020	114,146	640,306
2001	1,231,275	99,473	171,619	960,182
2002	2,440,344	217,813	349,767	1,872,764
2003	3,744,623	335,704	540,081	2,868,838
2004	4,869,883	426,452	704,260	3,739,172
2005	5,336,790	468,088	796,059	4,072,643
2006	6,975,220	633,107	1,014,292	5,327,821
2007	7,157,315	652,795	1,044,293	5,460,227
2008	7,501,516	671,090	1,110,240	5,720,185
2009	8,685,402	774,717	1,328,672	6,582,012
2010	9,763,080	878,663	1,438,974	7,445,443
2011	11,022,990	986,504	1,625,317	8,411,169
2012	10,930,176	941,730	1,598,470	8,389,975
2013	8,845,721	760,576	1,309,439	6,775,706
2014	9,343,998	837,266	1,328,696	7,178,036
2015	5,677,035	510,025	805,890	4,361,120
2016	5,615,207	492,931	800,411	4,321,865
2017	5,253,221	463,883	778,064	4,011,273
2018	5,317,697	487,172	769,136	4,061,389

Note:

[1] Patients' month and day of birth was imputed as July 1st for all patients. Age at onset was calculated as the difference between condition start date and imputed birth date.

Supplemental Table A3. Size of MarketScan Medicaid-insured population at risk in person-years (1998-2018)

	Total Population at risk in person years			
**Year**	**All ages**	**Age 0-1**	**Age 2-4**	**Age 5-17**
2001	1,309,359	236,937	261,527	810,895
2002	2,095,079	360,489	409,664	1,324,926
2003	2,453,468	400,734	483,844	1,568,890
2004	2,581,425	400,834	512,851	1,667,740
2005	2,813,872	436,038	566,058	1,811,776
2006	2,526,832	413,689	483,704	1,629,439
2007	2,406,917	408,134	456,321	1,542,461
2008	2,574,256	430,275	501,395	1,642,585
2009	2,983,346	479,269	614,240	1,889,837
2010	3,139,573	486,189	634,307	2,019,077
2011	2,898,033	426,211	581,710	1,890,111
2012	3,947,665	537,340	779,592	2,630,734
2013	4,032,490	531,923	787,962	2,712,605
2014	5,343,957	712,153	971,404	3,660,400
2015	5,722,235	732,848	1,000,325	3,989,062
2016	5,657,009	684,027	990,031	3,982,951
2017	5,652,255	673,614	1,019,899	3,958,741
2018	4,703,874	577,989	822,895	3,302,990

Note:
[1] Patients' month and day of birth was imputed as July 1st for all patients. Age at onset was calculated as the difference between condition start date and imputed birth date.

Supplemental Table A4. Demographic characteristics of the population at risk (1998-2018)

	**Commercially Insured**					**Medicaid**			
	**Pre-PCV7 (1998-1999)**	**Early PCV7 (2001-2005)**	**Late PCV7 (2006-2009)**	**Early PCV13 (2011-2013)**	**Late PCV13 (2014-2018)**	**Early PCV7 (2001-2005)**	**Late PCV7 (2006-2009)**	**Early PCV13 (2011-2013)**	**Late PCV13 (2014-2018)**
**Number of PY at risk, N**	**1,525,323**	**17,622,916**	**30,319,452**	**30,798,887**	**31,207,158**	**11,253,203**	**10,491,350**	**10,878,188**	**27,079,328**
**Number of children at risk, N**	**1,786,236**	**21,686,073**	**37,053,604**	**37,669,249**	**37,661,189**	**14,686,042**	**13,713,038**	**13,054,994**	**31,476,279**
**Age, mean (SD)**	9.36 (5.13)	9.17 (5.14)	9.10 (5.17)	9.11 (5.13)	9.18 (5.16)	7.90 (5.24)	7.95 (5.30)	8.13 (5.13)	8.58 (5.16)
0-1 year, n (%)	128,090 (8.4%)	1,547,530 (8.8%)	2,731,709 (9.0%)	2,688,810 (8.7%)	2,791,277 (8.9%)	1,835,031 (16.3%)	1,731,368 (16.5%)	1,495,474 (13.7%)	3,380,631 (12.5%)
2-4 years, n (%)	208,759 (13.7%)	2,561,786 (14.5%)	4,497,497 (14.8%)	4,533,226 (14.7%)	4,482,198 (14.4%)	2,233,944 (19.9%)	2,055,660 (19.6%)	2,149,265 (19.8%)	4,804,554 (17.7%)
5-17 years, n (%)	1,188,474 (77.9%)	13,513,600 (76.7%)	23,090,246 (76.2%)	23,576,851 (76.6%)	23,933,683 (76.7%)	7,184,228 (63.8%)	6,704,323 (63.9%)	7,233,450 (66.5%)	18,894,144 (69.8%)
**Male, n (%)**	781,982 (51.3%)	9,008,536 (51.1%)	15,486,019 (51.1%)	15,733,677 (51.1%)	15,933,139 (51.1%)	5,730,324 (50.9%)	5,341,057 (50.9%)	5,524,379 (50.8%)	13,847,263 (51.1%)
**Region**									
Northeast	236,547 (15.5%)	1,700,260 (9.6%)	3,280,655 (10.8%)	5,324,554 (17.3%)	5,597,806 (17.9%)	- -	- -	- -	- -
North Central	361,447 (23.7%)	3,855,309 (21.9%)	7,944,535 (26.2%)	7,389,842 (24.0%)	6,640,078 (21.3%)	- -	- -	- -	- -
South	671,600 (44.0%)	7,408,222 (42.0%)	14,189,698 (46.8%)	10,620,846 (34.5%)	12,769,811 (40.9%)	- -	- -	- -	- -
West	91,709 (6.0%)	4,415,513 (25.1%)	4,699,036 (15.5%)	6,651,946 (21.6%)	5,834,866 (18.7%)	- -	- -	- -	- -
Missing/unknown	164,020 (10.8%)	243,612 (1.4%)	205,529 (0.7%)	811,698 (2.6%)	364,597 (1.2%)	- -	- -	- -	- -
**Urbanicity**									
Urban, n (%)	337,203 (22.1%)	3,116,716 (17.7%)	4,802,730 (15.8%)	4,205,925 (13.7%)	3,543,028 (11.4%)	- -	- -	- -	- -
Rural, n (%)	1,023,769 (67.1%)	14,277,841 (81.0%)	25,345,433 (83.6%)	25,792,188 (83.7%)	26,288,879 (84.2%)	- -	- -	- -	- -
Missing	164,351 (10.8%)	228,359 (1.3%)	171,289 (0.6%)	800,774 (2.6%)	1,375,250 (4.4%)	- -	- -	- -	- -
**Health plan types**									
FFS, n (%)	506,524 (33.2%)	1,311,144 (7.4%)	624,969 (2.1%)	311,885 (1.0%)	485,225 (1.6%)	4,800,703 (42.7%)	3,313,709 (31.6%)	3,882,067 (35.7%)	9,330,480 (34.5%)
EPO, n (%)	7,713 (0.5%)	132,809 (0.8%)	216,797 (0.7%)	726,943 (2.4%)	336,806 (1.1%)	0 (0.0%)	0 (0.0%)	0 (0.0%)	0 (0.0%)
HMO, n (%)	133,900 (8.8%)	4,163,711 (23.6%)	4,836,348 (16.0%)	3,963,862 (12.9%)	3,173,868 (10.2%)	3,005,577 (26.7%)	6,174,475 (58.9%)	6,643,672 (61.1%)	17,688,874 (65.3%)
POS, n (%)	477,683 (31.3%)	2,613,797 (14.8%)	2,834,579 (9.3%)	1,904,164 (6.2%)	2,155,477 (6.9%)	3,446,166 (30.6%)	756,055 (7.2%)	343,209 (3.2%)	1,038 (0.0%)
PPO, n (%)	394,230 (25.8%)	8,730,598 (49.5%)	19,809,845 (65.3%)	19,222,249 (62.4%)	17,435,433 (55.9%)	0 (0.0%)	2,794 (0.0%)	0 (0.0%)	37,901 (0.1%)
CDHP, n (%)	0 (0.0%)	214,158 (1.2%)	820,817 (2.7%)	1,471,679 (4.8%)	3,286,131 (10.5%)	0 (0.0%)	0 (0.0%)	0 (0.0%)	0 (0.0%)
HDHP, n (%)	0 (0.0%)	0 (0.0%)	126,870 (0.4%)	1,446,072 (4.7%)	3,219,459 (10.3%)	0 (0.0%)	0 (0.0%)	0 (0.0%)	0 (0.0%)
Missing, n (%)	5,273 (0.3%)	456,699 (2.6%)	1,049,227 (3.5%)	1,752,033 (5.7%)	1,114,759 (3.6%)	757 (0.0%)	244,317 (2.3%)	9,239 (0.1%)	21,036 (0.1%)

Notes:
[1] Enrollees' month and day of birth was imputed as July 1st for all patients. Age at onset was calculated as the difference between condition start date and imputed birth date.
[2] Enrollees' demographic characteristics and risk factors were first determined by each calendar year and then combined by PCV periods, assuming each year has distinct enrollee population.
[3] Standard deviations for age in each vaccine period were calculated using the pooled standard deviation of the samples in relevant years.
[4] All values, except for age and number of individuals at risk are reported in person years.
Abbreviations: CDHP: Consumer directed health plan; EPO: Exclusive provider organization; FFS: Fee-for-service; HDHP: High-deductible health plan; HMO: Health maintenance organization; PCV: Pneumococcal conjugate vaccine; POS: Point of service; PPO: Preferred provider organization; SD: Standard deviation.

Supplemental Table A5. Demographic characteristics of Medicaid insured children aged < 18 years with AOM episodes, by PCV period (2001-2018)

	**Medicaid**			
	**Early PCV7 (2001-2005)**	**Late PCV7 (2006-2009)**	**Early PCV13 (2011-2013)**	**Late PCV13 (2014-2018)**
**Total number of children with AOM, N**	**1,999,282**	**1,863,802**	**1,947,259**	**3,758,547**
**Age, mean (SD)**	3.42 (3.98)	3.47 (3.98)	3.84 (4.04)	4.09 (4.21)
<2 years, %	44.7%	43.8%	38.2%	36.8%
2-4 years, %	27.1%	27.3%	28.8%	27.7%
5-17 years, %	28.2%	29.0%	33.0%	35.5%
**Male, %**	51.6%	52.0%	51.6%	51.7%
**Health plan types**				
HMO/EPO, %	22.3%	57.0%	61.9%	71.5%
PPO/POS, %	34.0%	8.4%	3.1%	0.1%
FFS, %	43.7%	32.5%	34.9%	28.3%
Missing, %	0.0%	2.0%	0.0%	0.1%

Notes:
[1] Patients' month and day of birth was imputed as July 1st for all patients. Age at onset was calculated as the difference between condition start date and imputed birth date.
[2] Patients' demographic characteristics and risk factors were firstly determined by each calendar year and then combined by PCV periods, assuming each year has a distinct patient population.
[3] For each calendar year, patients' demographic characteristics were determined at the index episode, which was defined as the first AOM episode in the given calendar year.
[4] Standard deviations for age in each vaccine period were calculated using the pooled standard deviation of the samples in relevant years.
Abbreviations: AOM: Acute otitis media; EPO: Exclusive provider organization; FFS: Fee-for-service; HMO: Health maintenance organization; PCV: Pneumococcal conjugate vaccine; POS: Point of service; PPO: Preferred provider organization; SD: Standard deviation.

Supplemental Table A6. Incidence of risk factors for pneumococcal disease among AOM patients aged <18 years in the 6 months prior to AOM episodes, 1998-2018

	Commercially Insured					Medicaid			
	**Pre-PCV7 (1998-1999)**	**Early PCV7 (2001-2005)**	**Late PCV7 (2006-2009)**	**Early PCV13 (2011-2013)**	**Late PCV13 (2014-2018)**	**Early PCV7 (2001-2005)**	**Late PCV7 (2006-2009)**	**Early PCV13 (2011-2013)**	**Late PCV13 (2014-2018)**
**Total number of AOM patients, N**	**178,263**	**1,527,052**	**3,022,308**	**2,899,874**	**3,248,907**	**1,335,071**	**1,321,804**	**1,509,851**	**3,161,210**
**Risk factors**									
Chronic heart disease, n (%)	243 (0.1%)	2,489 (0.2%)	5,490 (0.2%)	6,559 (0.2%)	10,583 (0.3%)	3,829 (0.3%)	4,421 (0.3%)	5,332 (0.4%)	15,514 (0.5%)
Chronic lung disease including asthma, n (%)	3,540 (2.0%)	29,465 (1.9%)	67,996 (2.2%)	77,522 (2.7%)	84,490 (2.6%)	55,799 (4.2%)	59,868 (4.5%)	77,640 (5.1%)	139,989 (4.4%)
Diabetes mellitus, n (%)	246 (0.1%)	2,849 (0.2%)	5,924 (0.2%)	6,164 (0.2%)	8,267 (0.3%)	2,943 (0.2%)	3,053 (0.2%)	4,098 (0.3%)	10,402 (0.3%)
Cerebrospinal fluid leaks, n (%)	2 (0.0%)	13 (0.0%)	35 (0.0%)	54 (0.0%)	51 (0.0%)	23 (0.0%)	55 (0.0%)	37 (0.0%)	64 (0.0%)
Cochlear implant(s), n (%)	9 (0.0%)	52 (0.0%)	149 (0.0%)	178 (0.0%)	221 (0.0%)	61 (0.0%)	79 (0.0%)	111 (0.0%)	256 (0.0%)
Sickle cell disease or other hemoglobinopathies, and anatomic or functional asplenia, n (%)	62 (0.0%)	377 (0.0%)	992 (0.0%)	2,997 (0.1%)	2,038 (0.1%)	1,508 (0.1%)	1,304 (0.1%)	2,217 (0.1%)	4,780 (0.2%)
Congenital or acquired immunodeficiency, n (%)	93 (0.1%)	1,074 (0.1%)	2,785 (0.1%)	3,465 (0.1%)	8,370 (0.3%)	2,081 (0.2%)	1,771 (0.1%)	2,245 (0.1%)	9,913 (0.3%)
HIV infection, n (%)	10 (0.0%)	59 (0.0%)	66 (0.0%)	63 (0.0%)	64 (0.0%)	311 (0.0%)	225 (0.0%)	121 (0.0%)	170 (0.0%)
Chronic renal failure or nephrotic syndrome, n (%)	94 (0.1%)	783 (0.1%)	1,963 (0.1%)	2,303 (0.1%)	1,555 (0.0%)	1,124 (0.1%)	1,201 (0.1%)	1,490 (0.1%)	1,474 (0.0%)
Cancer and iatrogenic immunosuppression, including radiation therapy, n (%)	2,759 (1.5%)	14,079 (0.9%)	27,331 (0.9%)	35,109 (1.2%)	49,028 (1.5%)	16,683 (1.2%)	19,498 (1.5%)	51,557 (3.4%)	158,829 (5.0%)
Solid organ transplant, n (%)	25 (0.0%)	271 (0.0%)	648 (0.0%)	785 (0.0%)	1,222 (0.0%)	1,256 (0.1%)	403 (0.0%)	542 (0.0%)	1,456 (0.0%)
Alcoholism, n (%)	4 (0.0%)	52 (0.0%)	123 (0.0%)	108 (0.0%)	261 (0.0%)	78 (0.0%)	60 (0.0%)	96 (0.0%)	541 (0.0%)
Chronic liver disease, n (%)	24 (0.0%)	177 (0.0%)	412 (0.0%)	433 (0.0%)	1,233 (0.0%)	308 (0.0%)	329 (0.0%)	417 (0.0%)	2,104 (0.1%)
Multiple myeloma, n (%)	0 (0.0%)	0 (0.0%)	0 (0.0%)	1 (0.0%)	0 (0.0%)	0 (0.0%)	0 (0.0%)	0 (0.0%)	1 (0.0%)
Tobacco use, n (%)	2 (0.0%)	39 (0.0%)	206 (0.0%)	457 (0.0%)	444 (0.0%)	158 (0.0%)	287 (0.0%)	745 (0.0%)	2,720 (0.1%)
Hearing loss, n (%)	21 (0.0%)	240 (0.0%)	847 (0.0%)	1,592 (0.1%)	2,314 (0.1%)	296 (0.0%)	676 (0.1%)	1,319 (0.1%)	3,506 (0.1%)
Pre-term birth, n (%)	271 (0.2%)	3,510 (0.2%)	4,486 (0.1%)	3,092 (0.1%)	5,390 (0.2%)	6,647 (0.5%)	4,938 (0.4%)	3,830 (0.3%)	10,682 (0.3%)

Notes:
[1] Patients' month and day of birth was imputed as July 1st for all patients. Age at onset was calculated as the difference between condition start date and imputed birth date.
[2] Patients' risk factors were firstly determined by each calendar year and then combined by PCV periods, assuming each year has distinct patient population.
[3] For each calendar year, the index episode was defined as the first AOM episode in the given calendar year.
[4] Patients were required to be continuously enrolled in the health plan for at least 6-months prior to the start of index episode.
[5] For each calendar year, risk factors were determined based on medical claims during the 6-months pre-index period.
Abbreviations: AOM: Acute otitis media; HIV: Human Immunodeficiency Virus; PCV: Pneumococcal conjugate vaccine.

Supplemental Figure A1. Annual incidence rates of AOM episodes in Medicaid insured children by age group, in episodes per 1,000 PY (2001-2018)

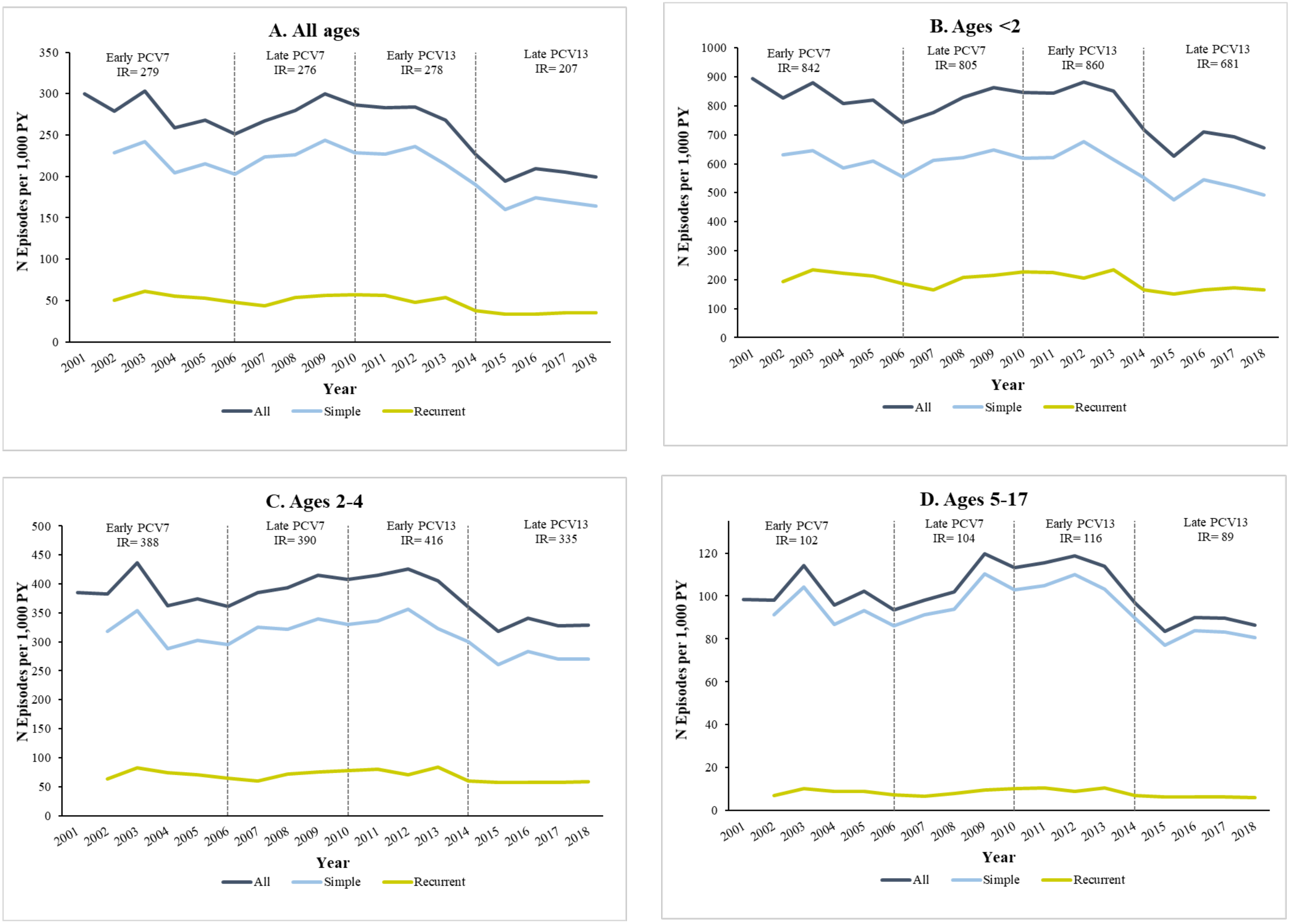

Note:
[1] Simple and recurrent episodes were categorized in patients with at least 12 months of continuous health plan enrollment prior to the index episode.
Abbreviations: AOM: Acute otitis media; PCV: Pneumococcal conjugate vaccine; PY: Person-years.

Supplemental Table A7. AOM episode incidence rates by study period for Medicaid insured children, in episodes per 1,000 PY (2001-2018)

	**All ages**			**Ages <2**		
**Period**	**Overall**	**Recurrent**	**Simple**	**Overall**	**Recurrent**	**Simple**
Early PCV7	279.5 (279.2; 279.8)	54.9 (54.8; 55.1)	221.9 (221.6; 222.1)	841.6 (840.3; 842.9)	216.1 (215.3; 216.8)	617.6 (616.4; 618.8)
Late PCV7	275.8 (275.5; 276.1)	50.7 (50.6; 50.9)	225.1 (224.8; 225.4)	804.9 (803.5; 806.2)	194.8 (194.1; 195.5)	610.1 (608.9; 611.2)
Early PCV13	277.6 (277.3; 278.0)	52.4 (52.3; 52.6)	225.2 (224.9; 225.5)	860.5 (859.0; 862.0)	221.5 (220.8; 222.3)	639.0 (637.7; 640.3)
Late PCV13	206.8 (206.6; 206.9)	35.2 (35.1; 35.2)	171.6 (171.4; 171.8)	680.7 (679.8; 681.6)	163.2 (162.8; 163.7)	517.5 (516.7; 518.3)
	**Ages 2-4**			**Ages 5-17**		
	**Overall**	**Recurrent**	**Simple**	**Overall**	**Recurrent**	**Simple**
Early PCV7	387.6 (386.8; 388.4)	73.2 (72.8; 73.6)	314.8 (314.0; 315.6)	102.3 (102.0; 102.5)	8.9 (8.8; 9.0)	93.9 (93.6; 94.1)
Late PCV7	390.3 (389.5; 391.2)	68.6 (68.2; 68.9)	321.8 (321.0; 322.6)	104.0 (103.8; 104.3)	8.1 (8.0; 8.1)	96.0 (95.8; 96.2)
Early PCV13	415.8 (414.9; 416.7)	77.6 (77.2; 78.0)	338.2 (337.4; 339.0)	116.1 (115.8; 116.3)	10.0 (9.9; 10.1)	106.1 (105.9; 106.3)
Late PCV13	335.1 (334.6; 335.6)	57.9 (57.7; 58.1)	277.3 (276.8; 277.7)	89.3 (89.2; 89.5)	6.5 (6.4; 6.5)	82.9 (82.7; 83.0)

Notes:

[1] Confidence intervals were calculated using the Pearson method.

[2] Time periods are defined as follows: Early PCV7: 2001-2005; Late PCV7: 2006-2009; Early PCV13: 2011-2013; Late PCV13: 2014-2018. Year 2010 is considered transition years and was excluded.

[3] Recurrent AOM was defined as having three or more episodes within a 6 months period or 4 or more episodes within a 12 month period, with at least one episode in the proceeding 6 months.

Abbreviations: AOM: Acute otitis media; PY: Person-years.

Supplemental Figure A2. AOM episode incidence rates stratified by sex for commercially insured children, in episodes per 1,000 PY (1998-2018)

A. By Year

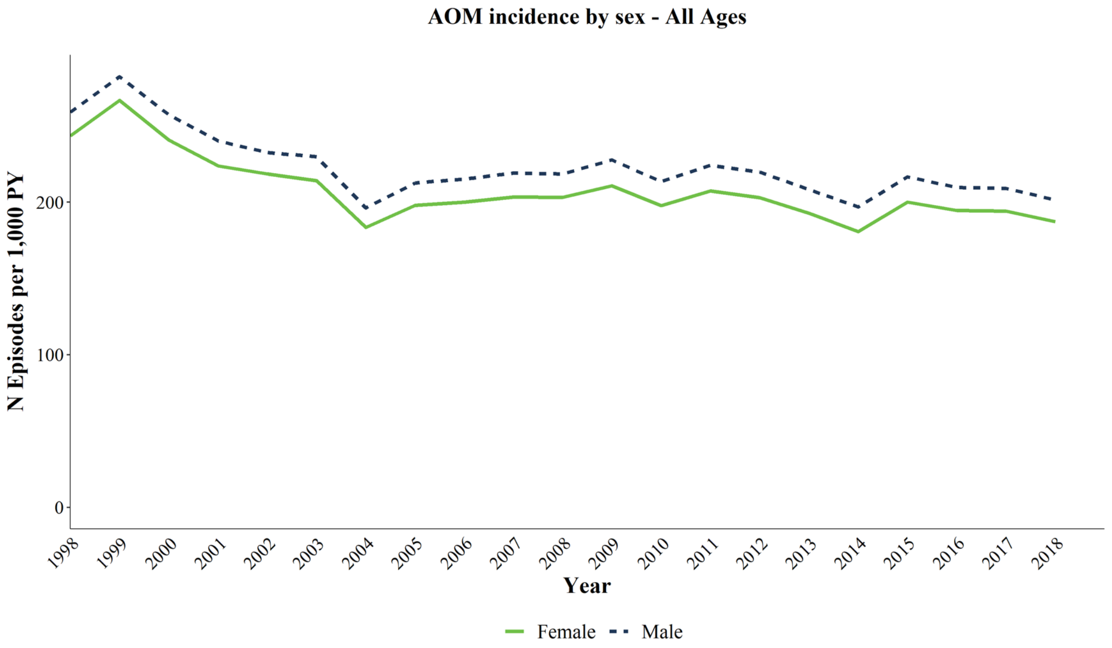

B. By Period

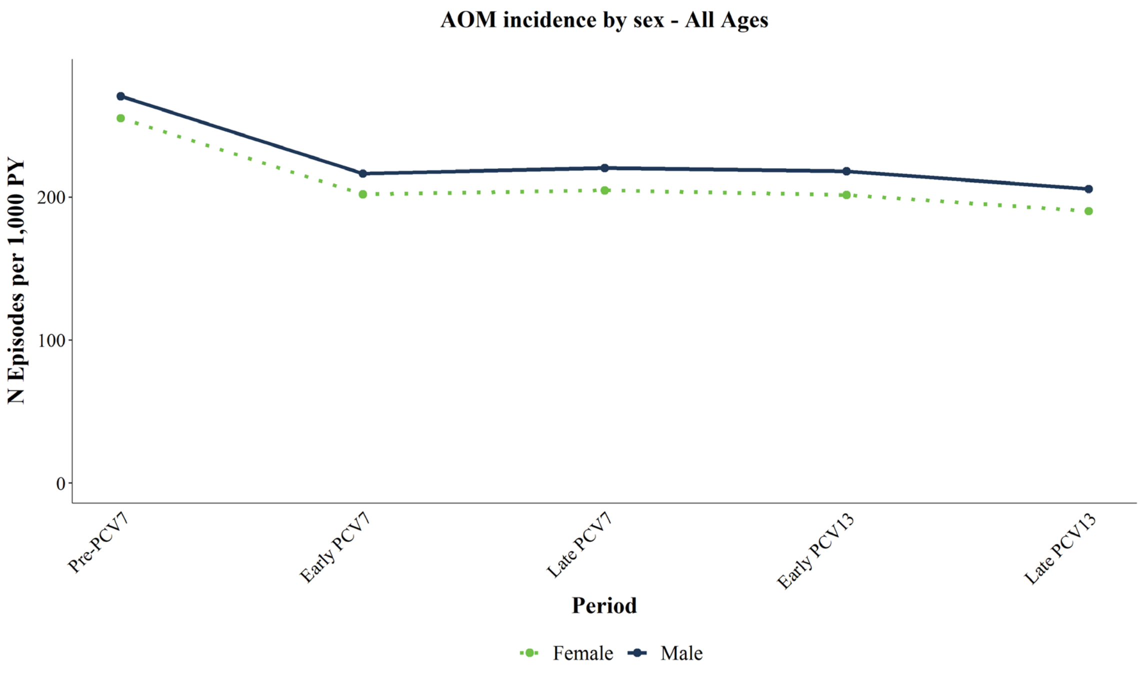

Notes:
[1] Patients' month and day of birth were imputed as July 1st for all patients. Age at onset was calculated as the difference between condition start date and imputed birth date.
[2] Time periods are defined as follows: Pre-PCV7: 1998-1999; Early PCV7: 2001-2005; Late PCV7: 2006-2009; Early PCV13: 2011-2013; Late PCV13: 2014-2018. Years 2000 and 2010 are considered transition years and were excluded.
Abbreviations: AOM: Acute otitis media; PY: Person-years.

Supplemental Figure A3. AOM episode incidence rates stratified by region for the commercially insured children, in episodes per 1,000 PY (1998-2018)

A. By Year

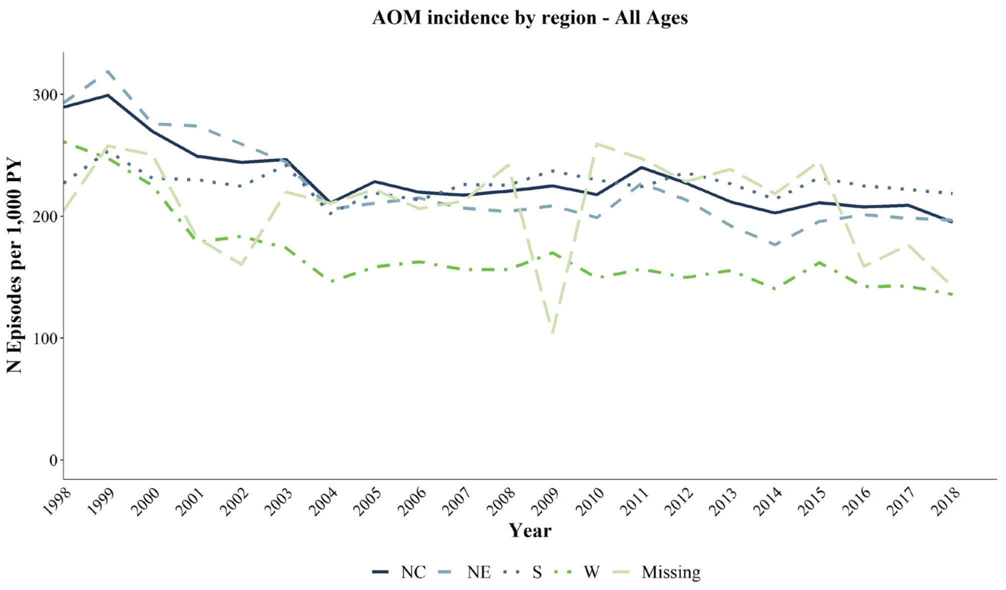

B. By Period

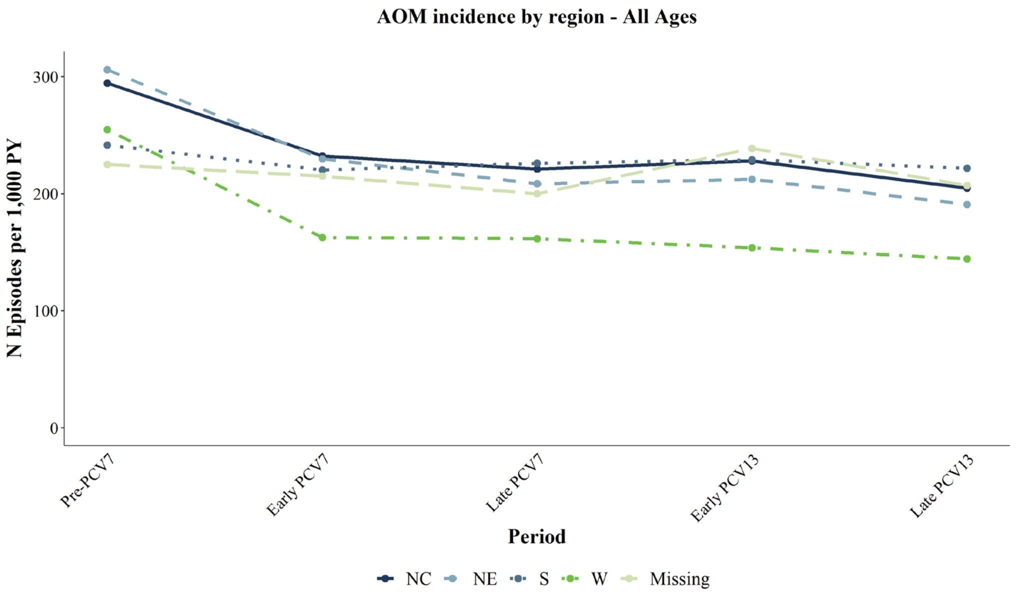

Notes:
[1] Patients' month and day of birth were imputed as July 1st for all patients. Age at onset was calculated as the difference between condition start date and imputed birth date.
[2] Time periods are defined as follows: Pre-PCV7: 1998-1999; Early PCV7: 2001-2005; Late PCV7: 2006-2009; Early PCV13: 2011-2013; Late PCV13: 2014-2018. Years 2000 and 2010 are considered transition years and were excluded.
Abbreviations: AOM: Acute otitis media; NC: North Central; NE: North East; PY: Person-years; S: South; W: West.

Supplemental Figure A4. AOM episode incidence rates stratified by urbanicity for the commercially insured children, in episodes per 1,000 PY (1998-2018)

A. By year

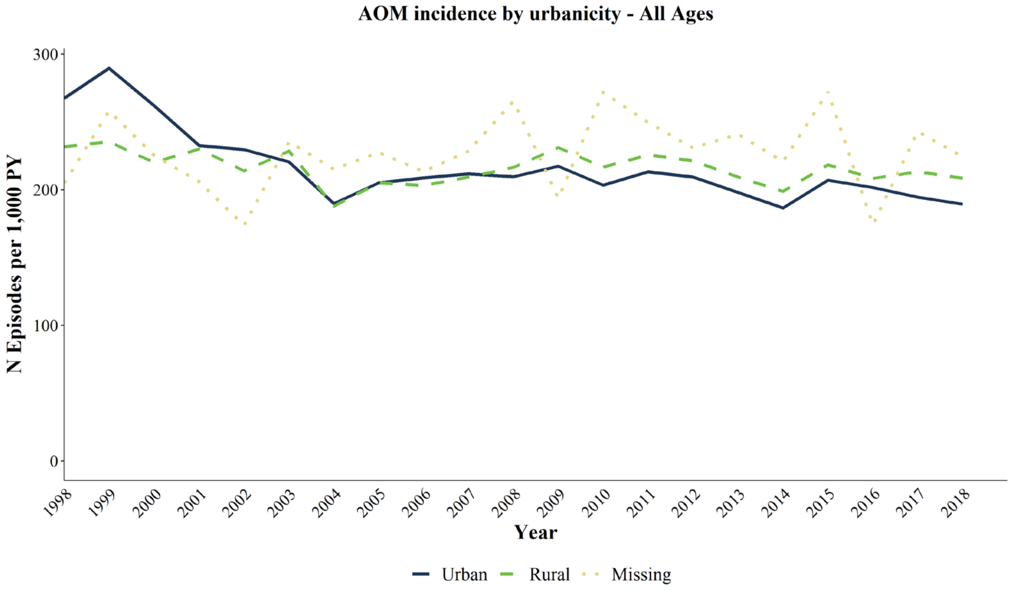

B. By period

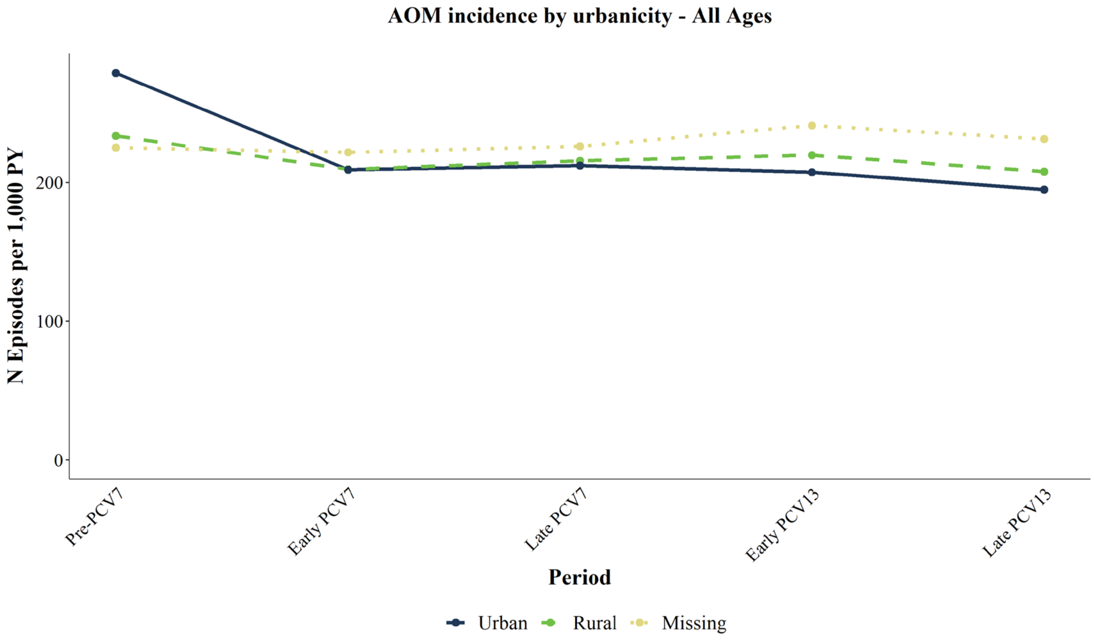

Notes:
[1] Patients' month and day of birth were imputed as July 1st for all patients. Age at onset was calculated as the difference between condition start date and imputed birth date.
[2] Time periods are defined as follows: Pre-PCV7: 1998-1999; Early PCV7: 2001-2005; Late PCV7: 2006-2009; Early PCV13: 2011-2013; Late PCV13: 2014-2018. Years 2000 and 2010 are considered transition years and were excluded.
Abbreviations: AOM: Acute otitis media; PY: Person-years.

Supplemental Figure A5. AOM episode incidence rates stratified by sex for Medicaid children, in episodes per 1,000 PY (2001-2018)

A. By year

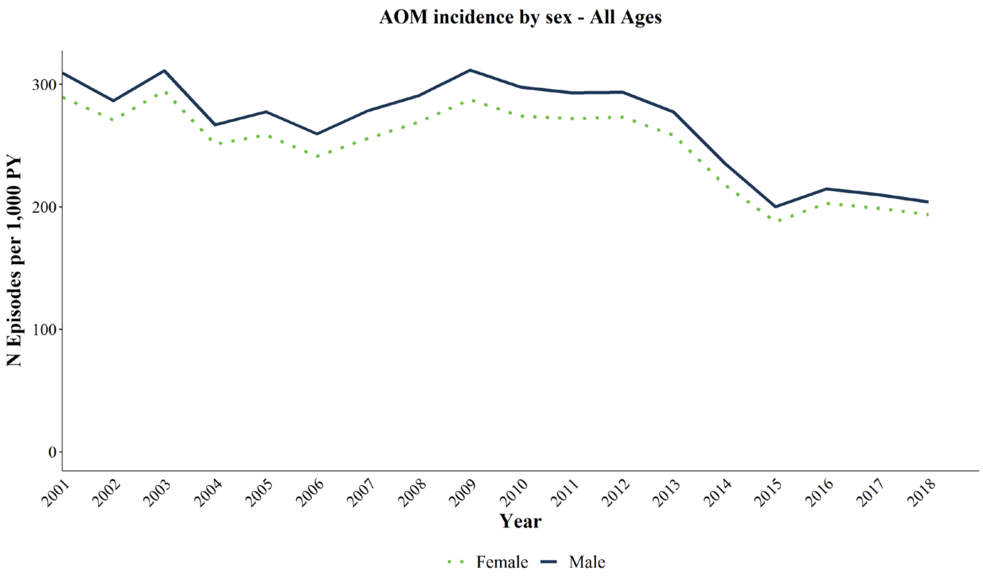

B. By period

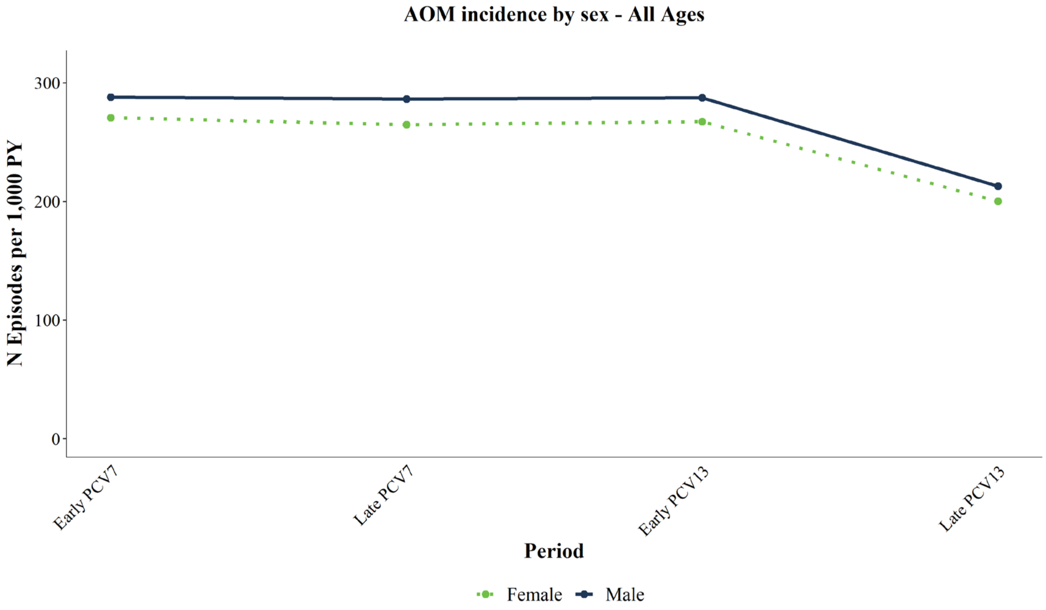

Notes:
[1] Patients' month and day of birth were imputed as July 1st for all patients. Age at onset was calculated as the difference between condition start date and imputed birth date.
[2] Time periods are defined as follows: Early PCV7: 2001-2005; Late PCV7: 2006-2009; Early PCV13: 2011-2013; Late PCV13: 2014-2018. Years 2000 and 2010 are considered transition years and were excluded.
Abbreviations: AOM: Acute otitis media; PY: Person-years

Supplemental Figure A6. Monthly incidence rates and linear time trends predicted from the ITS models in the commercially insured population aged <18 years, in episodes per 1,000 PY (1998-2018)

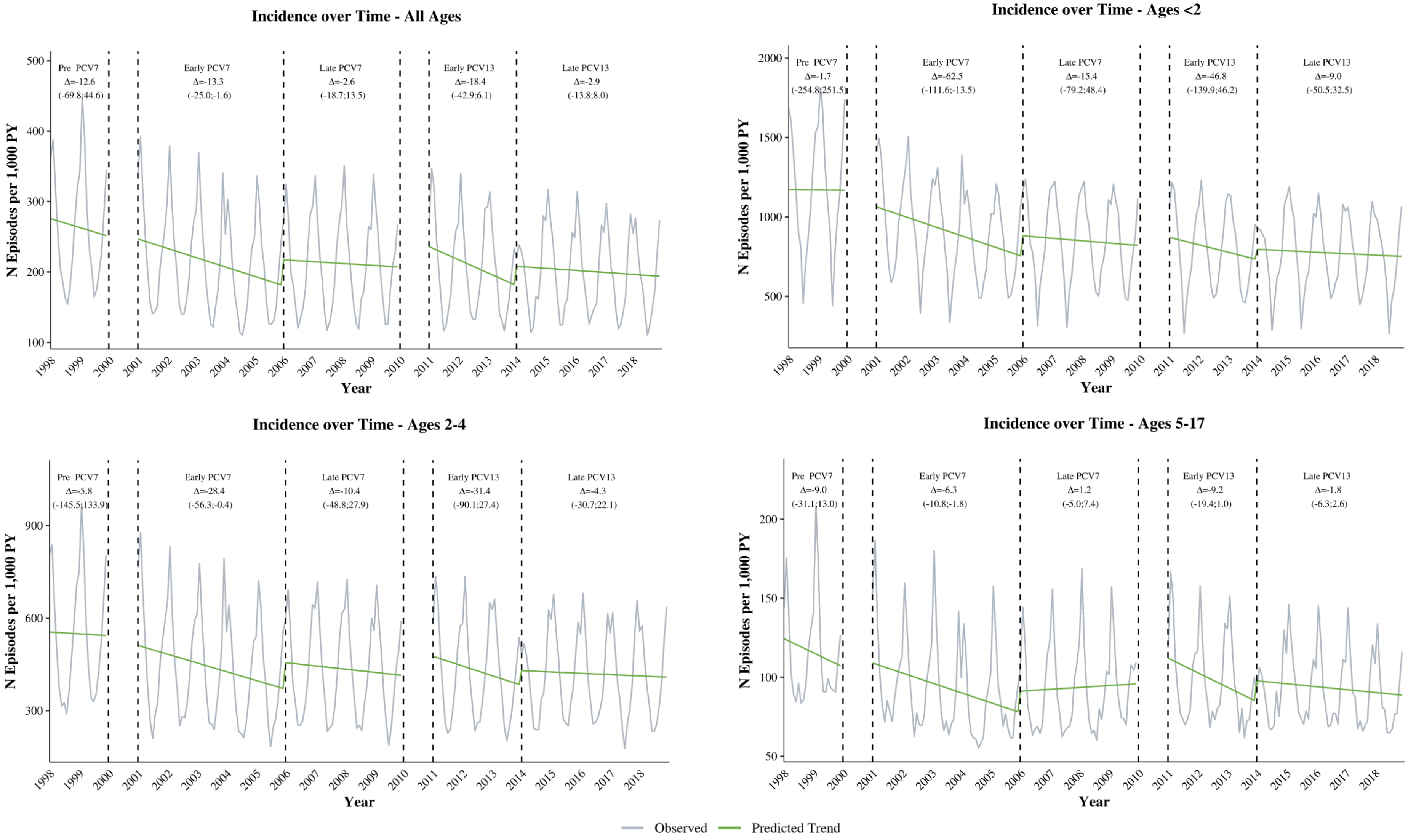

Notes:
[1] Predicted values for episode counts were obtained using a negative binomial GLM model with a log link, controlling for seasonality using monthly indicators. These values were then used to calculate linear trends in incidence rates (green lines). Observed monthly incidence rates are shown in gray.
[2] Time periods are defined as follows: Pre-PCV7: 1998-1999; Early PCV7: 2001-2005; Late PCV7: 2006-2009; Early PCV13: 2011-2013; Late PCV13: 2014-2018. Years 2000 and 2010 are considered transition years and were excluded from the model.
Abbreviations: AOM: Acute otitis media; GLM: Generalized linear model; PCV: Pneumococcal conjugate vaccine; PY: Person-years.

Supplemental Table A8. Estimates from interrupted time series analyses of monthly AOM episode incidence rates in Medicaid children <18 years (1998-2018)

		All ages		Ages <2		Ages 2-4		Ages 5-17	
**Period**	**Change**	**IRR (95% CI)**	***P***	**IRR (95% CI)**	***P***	**IRR (95% CI)**	***P***	**IRR (95% CI)**	***P***
Late PCV7	Base Trend	1.005 (1.004 - 1.006)	0.001*	1.004 (1.003 - 1.006)	0.001*	1.004 (1.003 - 1.005)	0.001*	1.007 (1.004 - 1.009)	0.001*
Early PCV13	Change in Level	0.993 (0.887 - 1.111)	0.902	0.984 (0.885 - 1.094)	0.765	1.034 (0.909 - 1.175)	0.614	1.016 (0.857 - 1.205)	0.851
	Change in Trend	0.992 (0.989 - 0.995)	0.001*	0.996 (0.992 - 0.999)	0.010*	0.995 (0.991 - 0.998)	0.003*	0.992 (0.987 - 0.997)	0.001*
Late PCV13	Change in Level	0.830 (0.727 - 0.947)	0.006*	0.810 (0.735 - 0.893)	0.001*	0.865 (0.762 - 0.982)	0.025*	0.828 (0.711 - 0.964)	0.015*
	Change in Trend	1.001 (0.997 - 1.005)	0.550	1.000 (0.996 - 1.003)	0.945	1.000 (0.996 - 1.005)	0.862	1.000 (0.995 - 1.005)	0.917

Notes:
[1] * Coefficients statistically significant at $P<0.05$.
[2] All estimates were obtained through a negative binomial model with a log link, controlling for seasonality using monthly indicators. IRR coefficients show the exponentiated regression results and represent a multiplicative change. Model intercepts not shown.
[3] Confidence intervals have been adjusted for heteroscedasticity.
[4] Time periods are defined as follows: Late PCV7: 2006-2009; Early PCV13: 2011-2013; Late PCV13: 2014-2018. Year 2010 is considered a transition year and was excluded from the model.
Abbreviations: AOM: Acute otitis media; IRR: Incidence rate ratio; PCV: Pneumococcal conjugate vaccine.

Supplemental Figure A7. Monthly incidence rates and linear time trends predicted from the ITS models in the Medicaid insured population aged <18 years, in episodes per 1,000 PY (2006-2018)

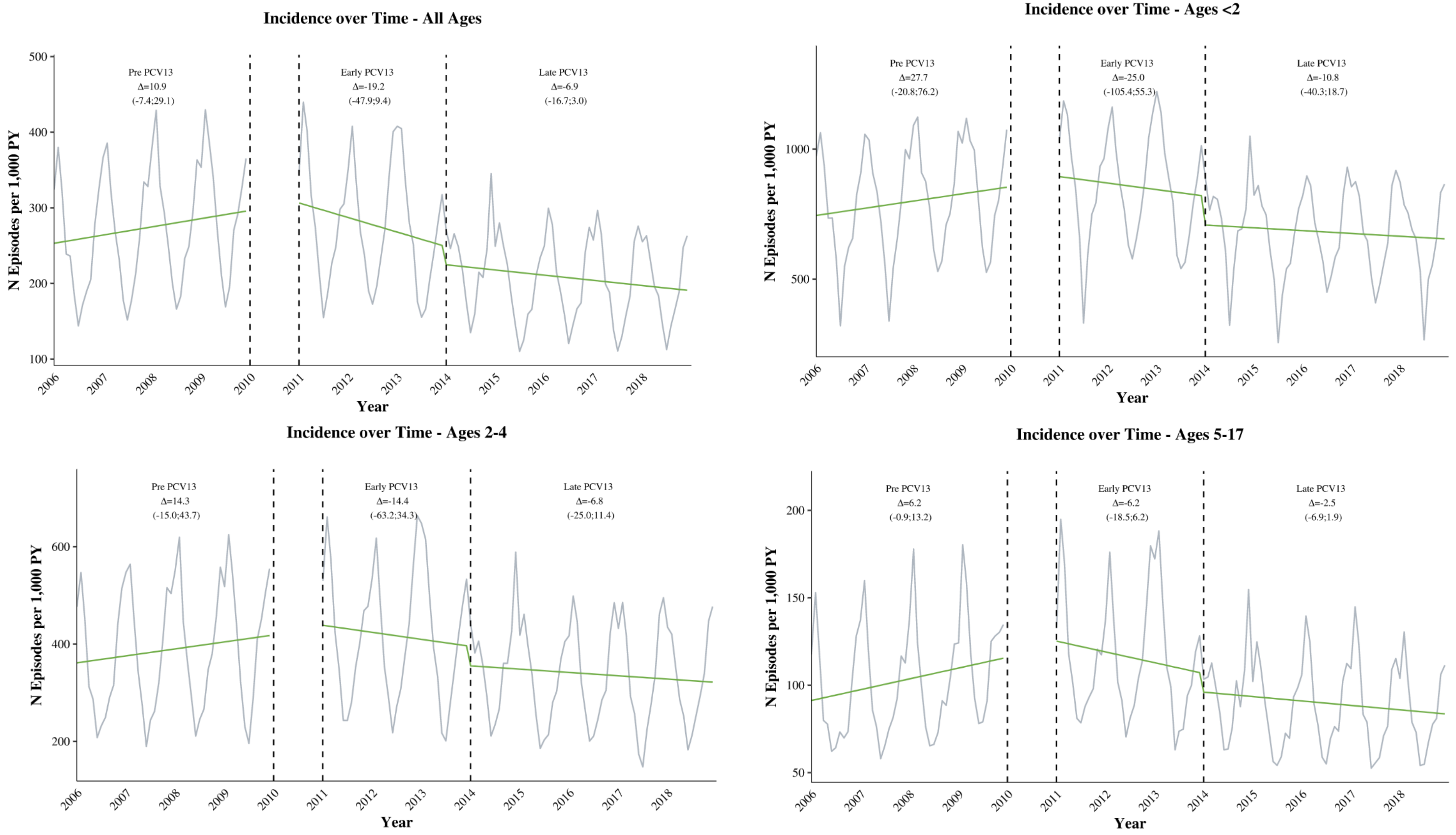

Notes:
[1] Predicted values for episode counts were obtained using a negative binomial GLM model with a log link , controlling for seasonality using monthly indicators. These values were then used to calculate linear trends in incidence rates (green lines). Observed monthly incidence rates are shown in gray.
[2] Time periods are defined as follows: Late PCV7: 2006-2009; Early PCV13: 2011-2013; Late PCV13: 2014-2018. Year 2010 is considered transition year and was excluded from the model.
Abbreviations: AOM: Acute otitis media; GLM: Generalized linear model; PCV: Pneumococcal conjugate vaccine; PY: Person-years.

Supplemental Figure A8. Annual incidence rates of AOM-related complications by age group among Medicaid insured children aged <18 years, in complications per 1,000 PY (1998-2018)

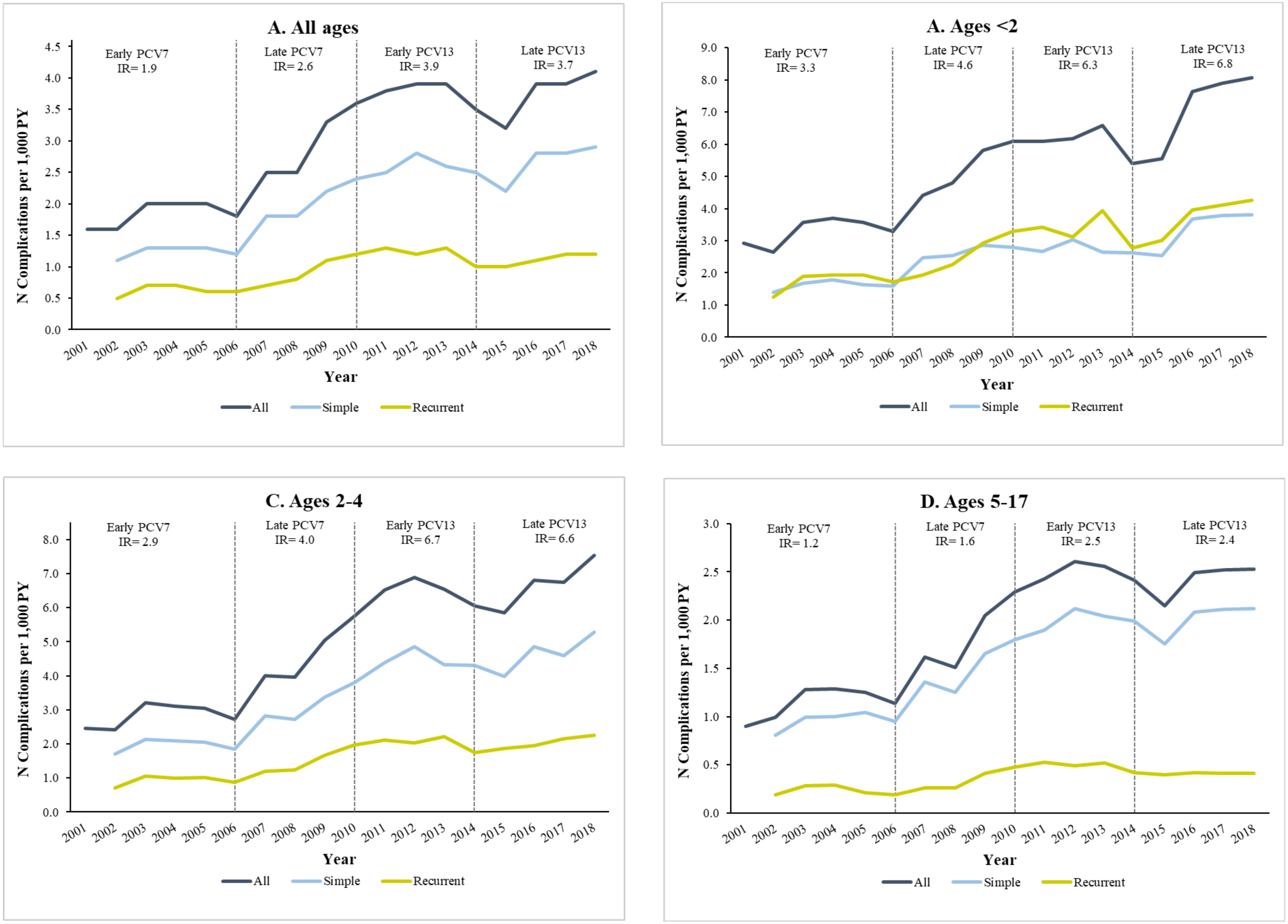

Supplemental Figure A9. Incidence rates of AOM-related complications by study period for the commercially insured and Medicaid children, in complications per 1,000 PY (1998-2018)

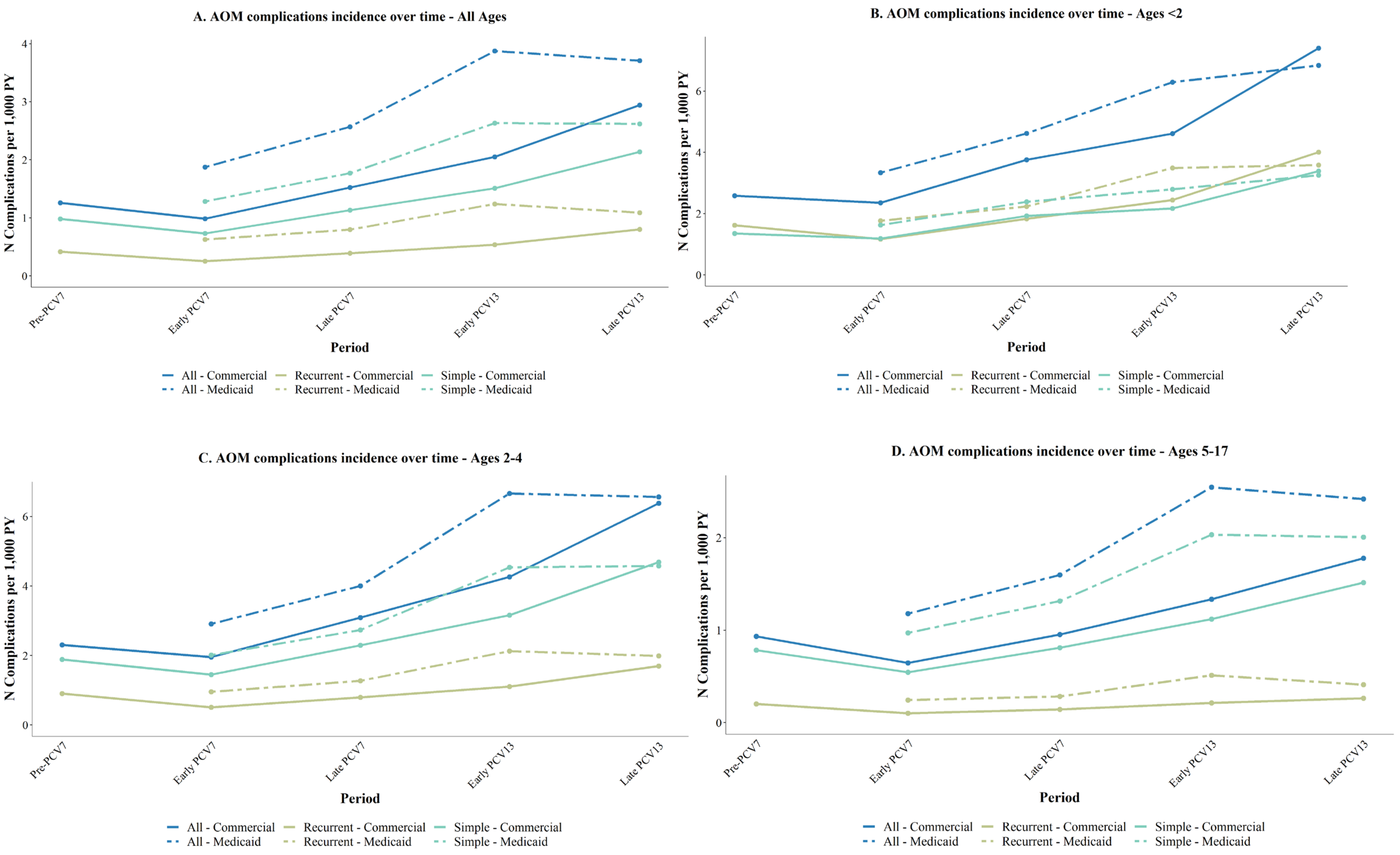

Notes:
[1] Patients' month and day of birth were imputed as July 1st for all patients. Age at onset was calculated as the difference between condition start date and imputed birth date.
[2] Time periods are defined as follows: Pre-PCV7:1998-1999; Early PCV7: 2001-2005; Late PCV7: 2006-2009; Early PCV13: 2011-2013; Late PCV13: 2014-2018. Years 2000 and 2010 are considered transition years and were excluded.
Abbreviations: AOM: Acute otitis media; PY: Person-years.

Supplemental Figure A10. Annual incidence rates of AOM-related surgical procedures by age group among Medicaid-insured children aged <18 years, in procedures per 1,000 PY (1998-2018)

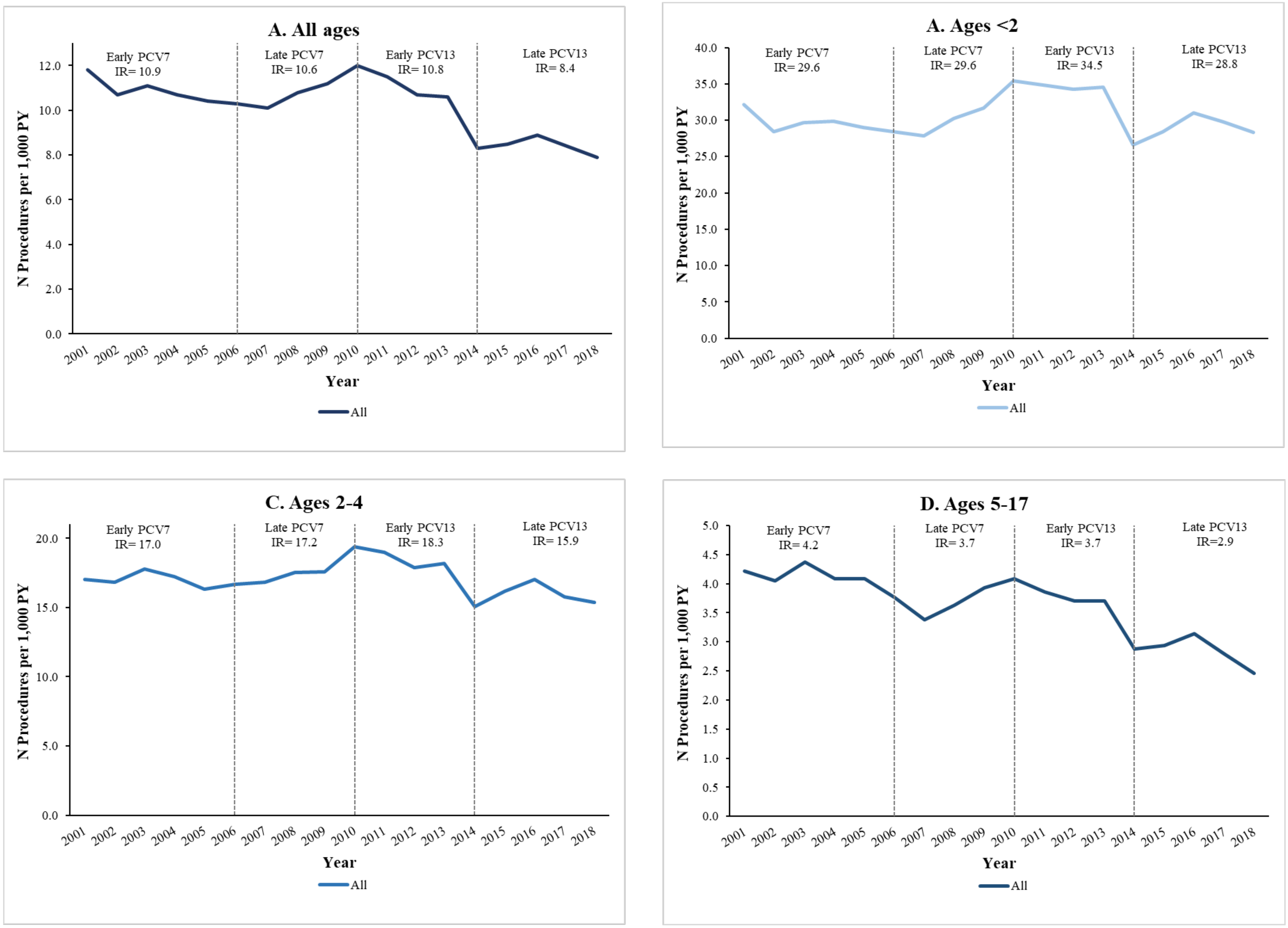

Note: Period-specific IRs are shown for total AOM-related procedures. **Abbreviations:** AOM: Acute otitis media; PY: Person-years.

Supplemental Figure A11. Incidence rates of AOM-related surgical procedures by study period for the commercially insured and Medicaid children, in procedures per 1,000 PY (1998-2018)

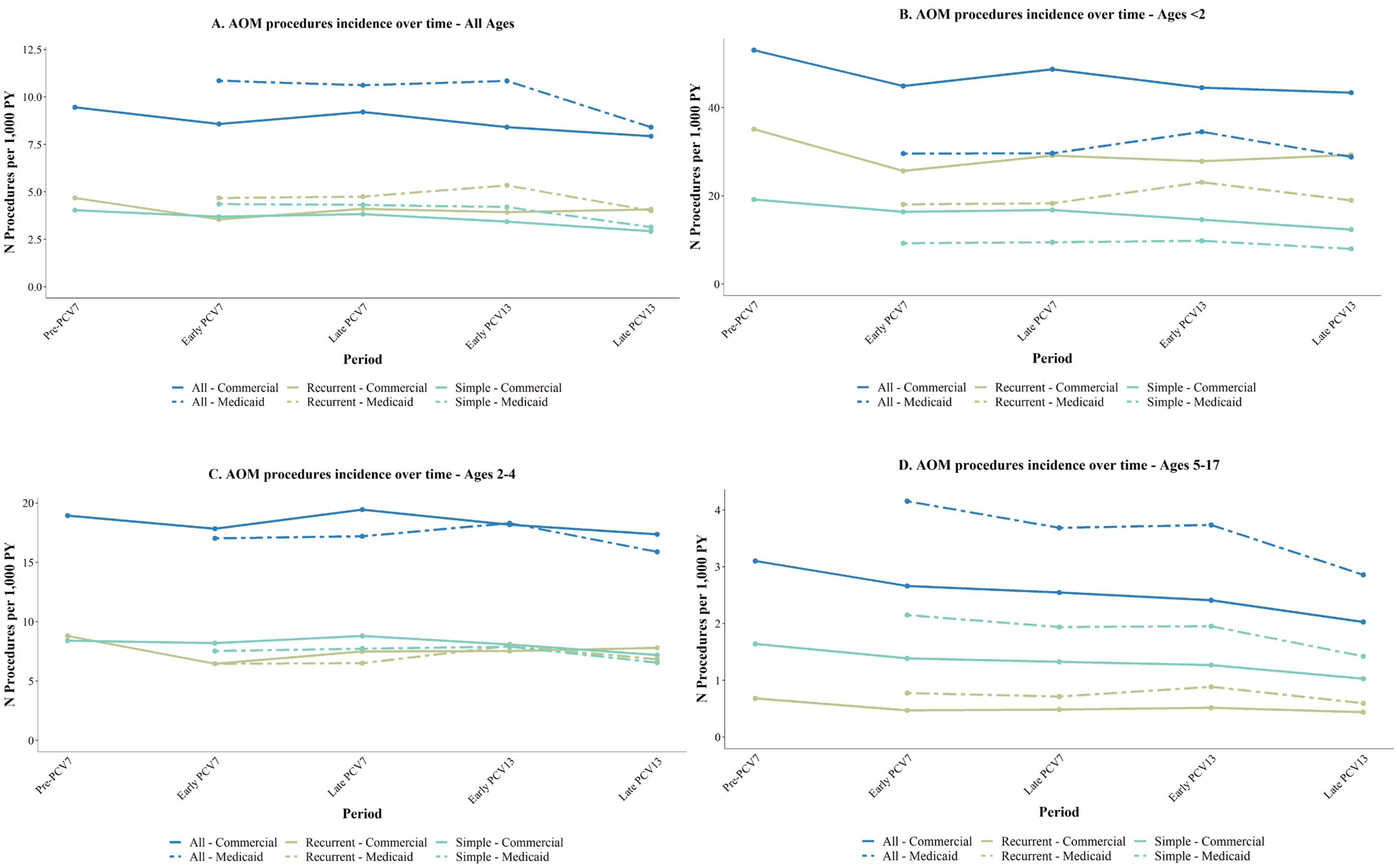

Notes:
[1] Patients' month and day of birth were imputed as July 1st for all patients. Age at onset was calculated as the difference between condition start date and imputed birth date.
[2] Time periods are defined as follows: Pre-PCV7:1998-1999; Early PCV7: 2001-2005; Late PCV7: 2006-2009; Early PCV13: 2011-2013; Late PCV13: 2014-2018. Years 2000 and 2010 are considered transition years and were excluded.
Abbreviations: AOM: Acute otitis media; PY: Person-years.

Supplemental Figure A12. Trends in annual incidence rates of AOM (28-day gap) by age group among children 0-17 years, in episodes per 1,000 PY (1998-2018)

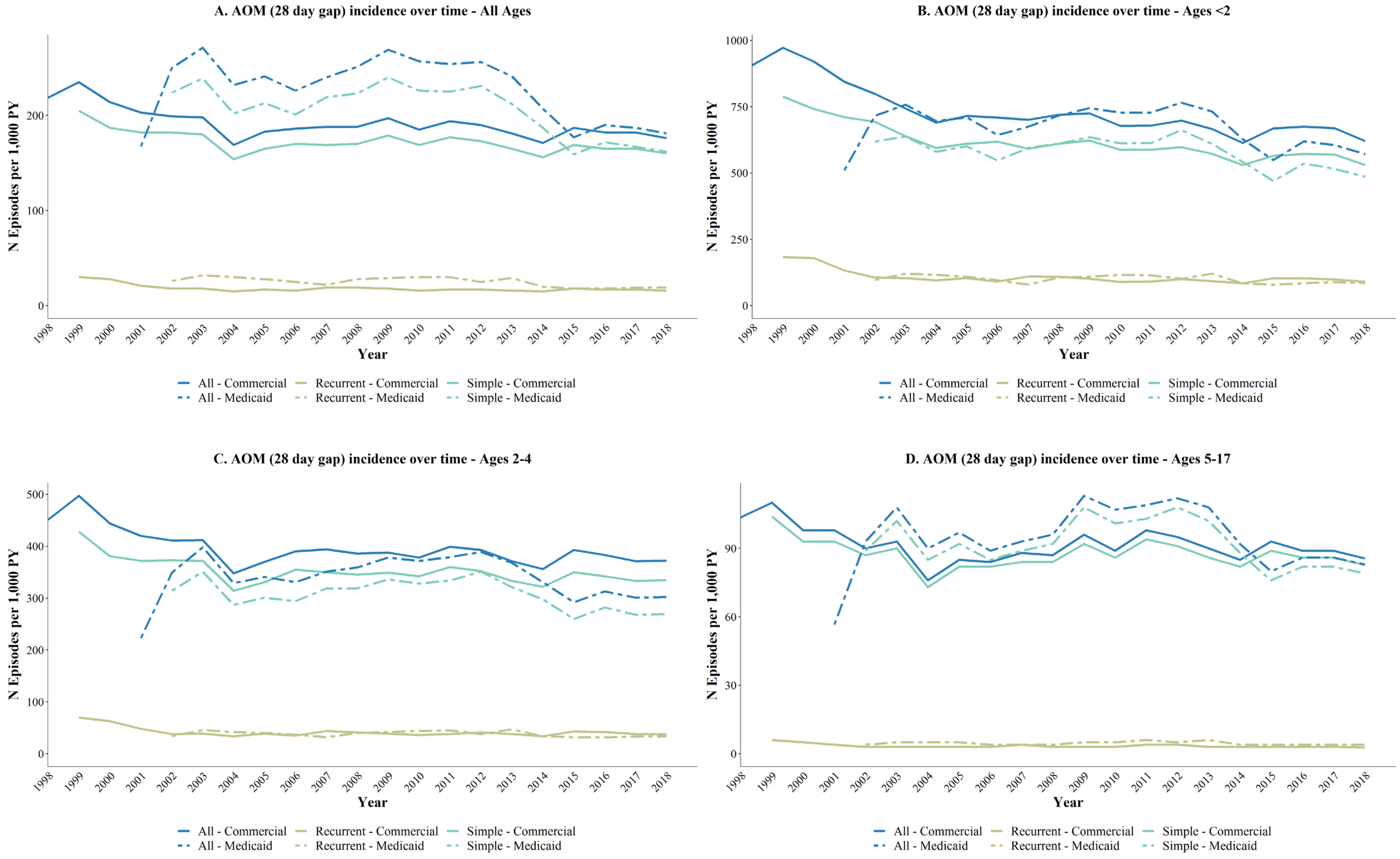

Abbreviations: AOM: Acute otitis media, PY: Person-years.

Supplemental Figure A13. Trends in period-specific incidence rates of AOM (28-day gap) by age group among children 0-17 years, in episodes per 1,000 PY (1998-2018)

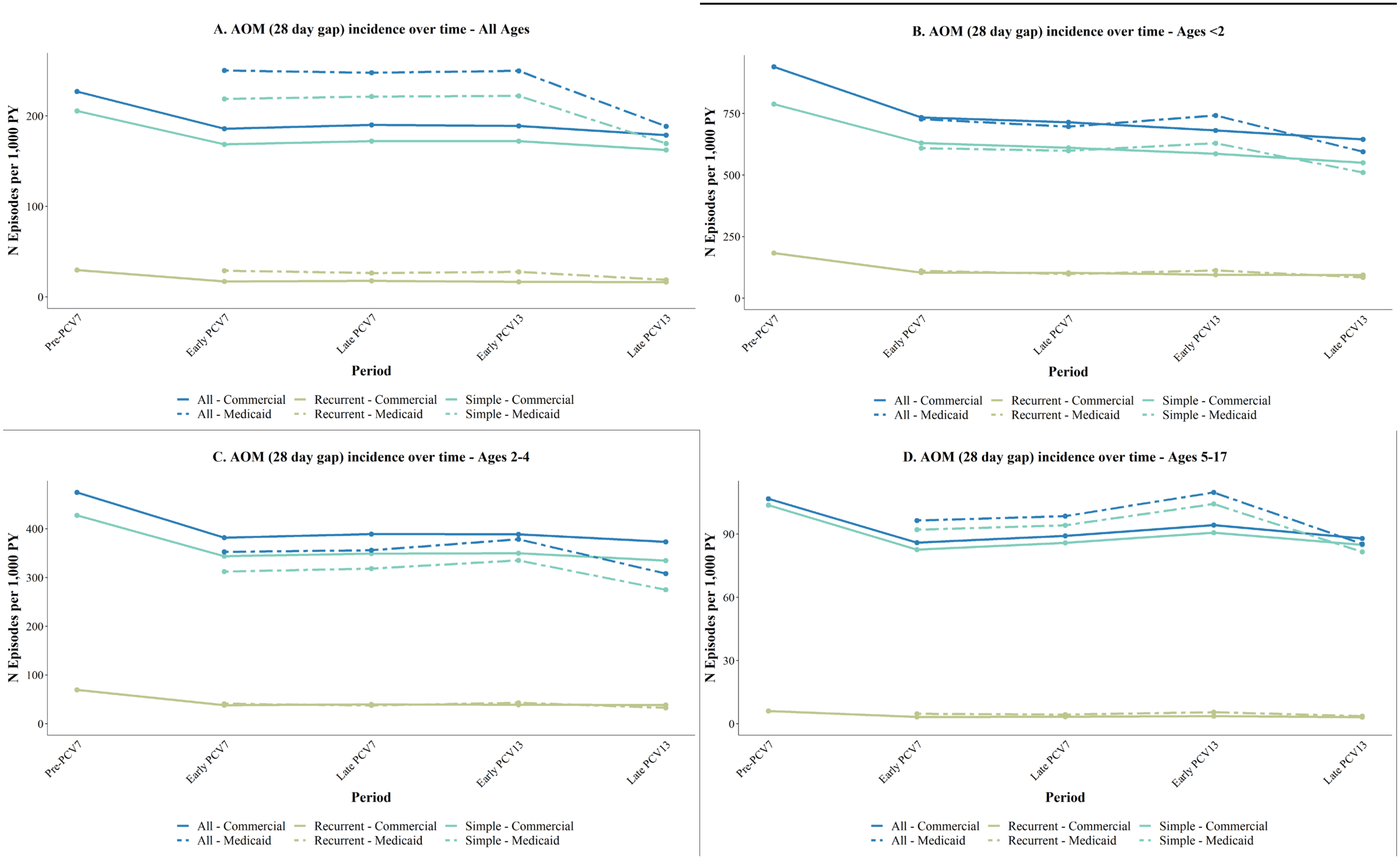

Notes:
[1] Patients' month and day of birth were imputed as July 1st for all patients. Age at onset was calculated as the difference between condition start date and imputed birth date.
[2] Time periods are defined as follows: Pre-PCV7: 1998-1999; Early PCV7: 2001-2005; Late PCV7: 2006-2009; Early PCV13: 2011-2013; Late PCV13: 2014-2018. Years 2000 and 2010 are considered transition years and were excluded.
Abbreviations: AOM: Acute otitis media; PY: Person-years

Supplemental Figure A14. Annual incidence rates of AOM + OME episodes among US children aged 0-17 years, in episodes per 1,000 PY (1998-2018)

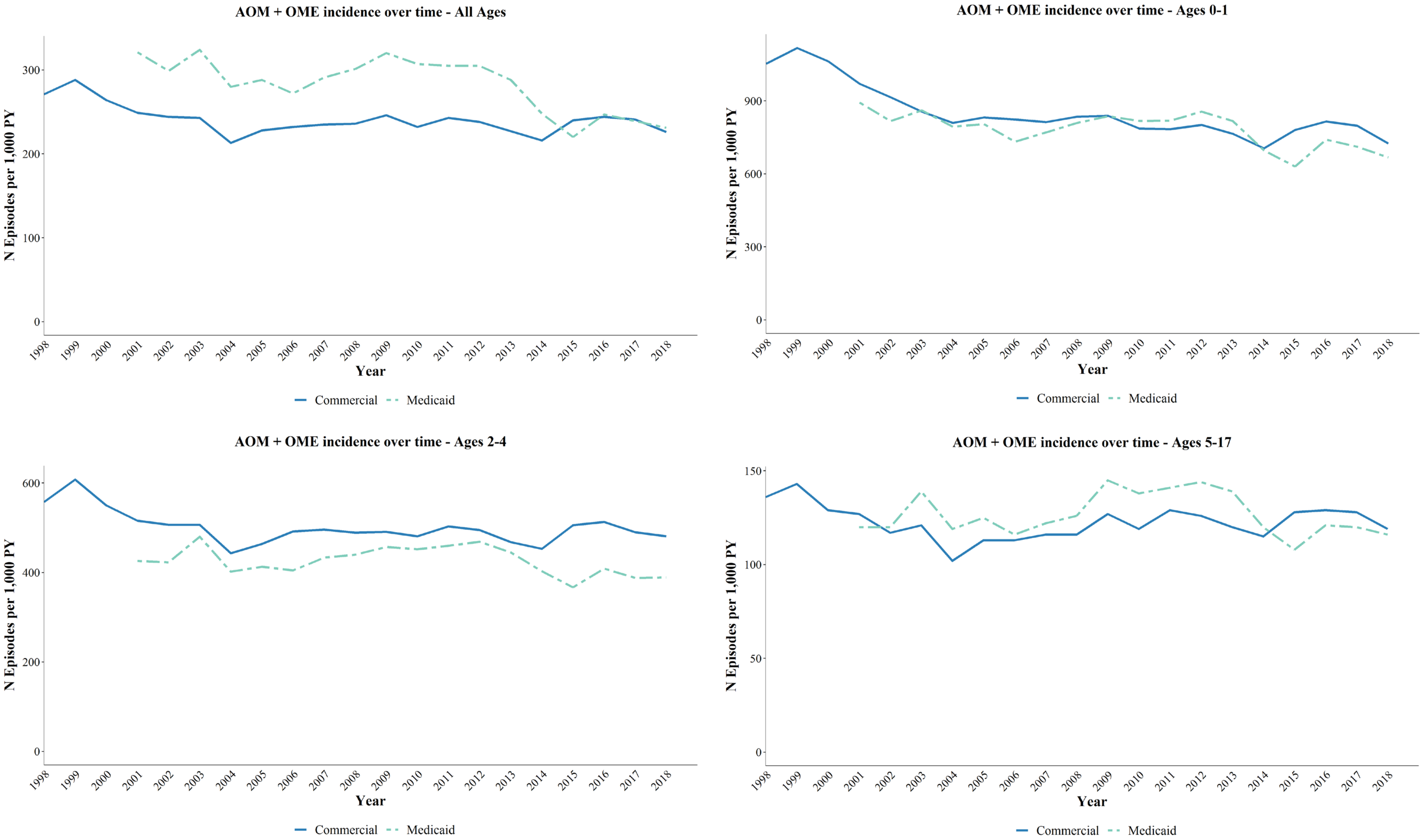

Notes:
[1] Patients' month and day of birth were imputed as July 1st for all patients. Age at onset was calculated as the difference between condition start date and imputed birth date.
Abbreviations: AOM: Acute otitis media, OME: Otitis media with effusion; PY: Person-years.

Supplemental Figure A15. Period-specific incidence rates of AOM + OME episodes for US children aged 0-17 years, in episodes per 1,000 PY (1998-2018)

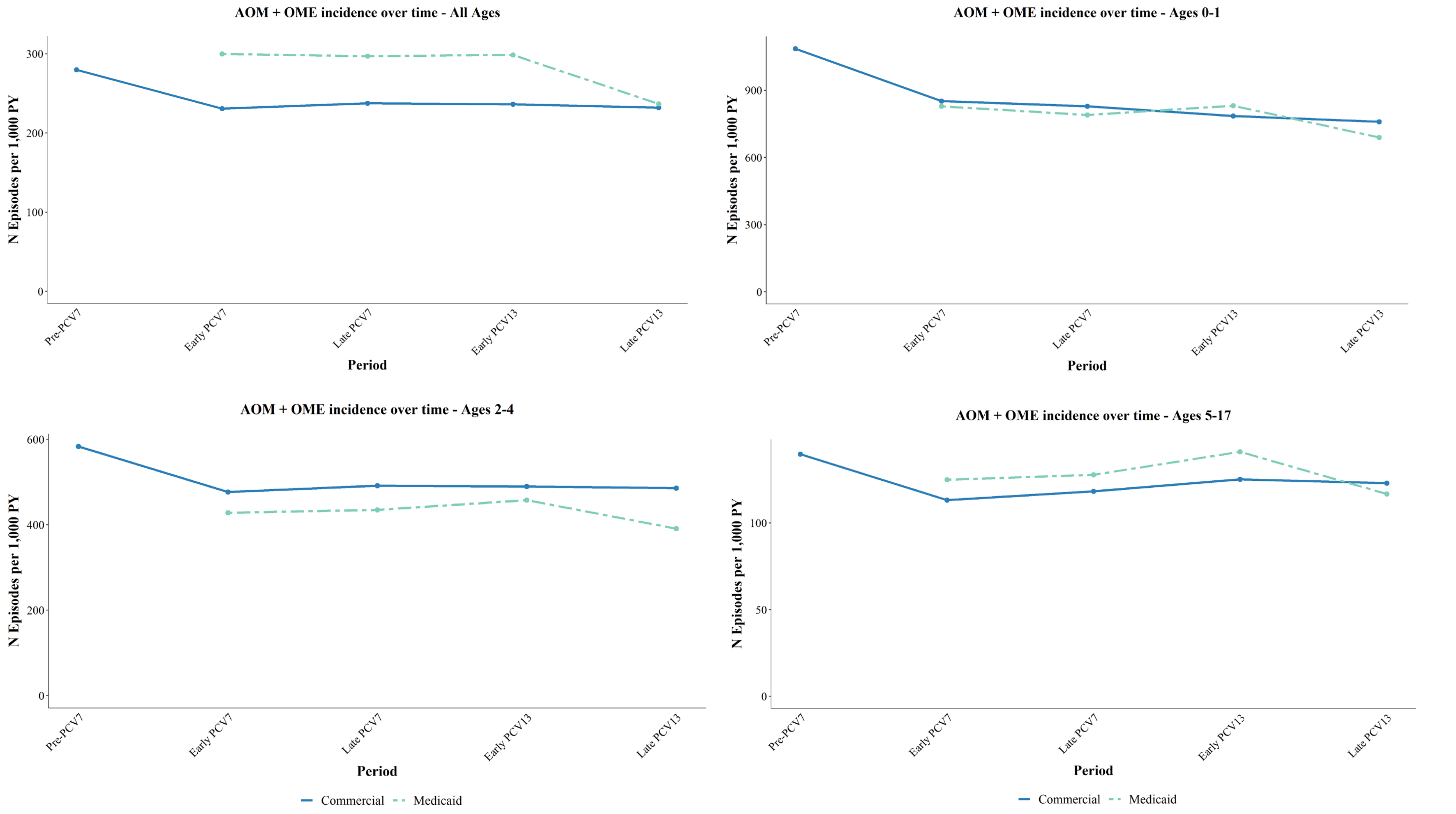

Notes:
[1] Patients' month and day of birth were imputed as July 1st for all patients. Age at onset was calculated as the difference between condition start date and imputed birth date.
[2] Time periods are defined as follows: Pre-PCV7: 1998-1999; Early PCV7: 2001-2005; Late PCV7: 2006-2009; Early PCV13: 2011-2013; Late PCV13: 2014-2018. Years 2000 and 2010 are considered transition years and were excluded.
Abbreviations: AOM: Acute otitis media, OME: Otitis media with effusion; PY: Person-years

Supplemental Table A9. Number of AOM episodes by ICD-9 codes, 1998-2015

	ICD-9 Code		
Year	**381.x:** Nonsuppurative otitis media and eustachian tube disorders	**382.x:** Suppurative and unspecified otitis media	**384.0x:** Acute myringitis without mention of otitis media
1998	47,429	158,352	225
1999	50,431	174,671	278
2000	49,937	169,430	306
2001	67,630	241,207	451
2002	129,076	470,692	810
2003	199,412	716,088	1,265
2004	251,521	794,808	1,468
2005	285,302	942,346	1,835
2006	385,494	1,249,000	2,375
2007	400,258	1,298,392	2,593
2008	422,413	1,362,270	2,824
2009	507,780	1,648,971	3,677
2010	554,515	1,739,601	3,778
2011	641,230	2,066,372	4,579
2012	624,712	2,009,155	4,505
2013	489,916	1,544,636	3,489
2014	506,244	1,543,034	3,642
2015	258,366	767,995	2,057
